# Activation parenting in mothers and fathers: A systematic review and meta-analysis

**DOI:** 10.1002/imhj.70101

**Published:** 2026-07

**Authors:** Dominic Laquerre, Amélie Gagné, Laurence Beaulieu, Mahée Gariépy, Julia Feldman, Jean-François Bureau, Audrey-Ann Deneault

**Affiliations:** 1 Département de psychologie, Université de Montréal, Montréal, Canada; 2 Centre de recherche, Institut universitaire en santé mentale de Montréal, Montréal, Canada; 3 Department of Psychiatry, University of Pittsburgh, Pittsburgh, USA; 4 School of Psychology, University of Ottawa, Ottawa, Canada

**Keywords:** activation parenting, challenging parenting behaviors, rough-and-tumble play, meta-analysis, systematic review

## Abstract

Evolutionary-based theories of parenting, such as activation relationship theory, suggest that fathers may be more likely than mothers to engage in activation parenting, that is, interactions designed to stimulate and challenge children within acceptable limits. However, available evidence is mixed in identifying differences between mothers and fathers in activation parenting. Using two meta-analyses, we examined 1) mean differences and 2) associations in activation parenting exhibited by partnered mothers and fathers. The meta-analyses included 40 studies (38 unique samples, 76.3% collected in North America) with a total of 3,607 fathers and 3,707 mothers, and their children (*M* = 41.4 months; range = 3 months to 19.9 years). Most parents were white and from a mid-high socioeconomic status. The first meta-analysis showed that fathers displayed higher activation parenting than mothers, with a small effect size: *g* = .27, 95% CI [.17, .37], *p* < .001. Differences were larger in studies examining rough-and-tumble play. A strong correlation between partnered parents’ activation parenting levels emerged in the second meta-analysis: *r* = .33, 95% CI [.19, .45], *p* < .001. These results highlight the importance of continuing research on activation parenting across diverse types of parents.

## 1 | INTRODUCTION

Much of parenting research is concerned with parents’ roles in fostering positive child development. Traditionally, this research has paid special attention to maternal sensitivity as a key driver of child well-being. Sensitive caregivers are characterized by their responsive, prompt, and adequate response to their children’s signals, which reflects their capacity to correctly interpret and fulfill their child’s needs (Ainsworth, 1969). While extensive research has shown that parental sensitivity is associated with various positive child outcomes (e.g., Cooke et al., 2022; Madigan et al., 2024; Rodrigues et al., 2021), a developing body of research has sought to expand our view of parenting behaviors beyond parental sensitivity, notably by examining activation parenting. Based on Paquette’s (2004a; Paquette & Puentes-Neuman, 2024) activation relationship theory, activation parenting includes behaviors that challenge and stimulate children beyond their comfort zone, either in a physical or socioemotional manner (Feldman & Shaw, 2021). Activation relationship theory suggests a gendered approach to understanding these parental behaviors, such that between opposite-gendered coparents, mothers are more likely to be sensitive, while fathers are more likely to be activating (Paquette, 2004a, 2004b; Paquette & Puentes-Neuman, 2024). Activation relationship theory shares a view with other theories that evolutionary forces brought a level of specialization in parental behaviors among fathers and mothers (e.g., Bögels & Perotti, 2011; Grossmann et al., 2002). In an empirical test of this hypothesis, a recent meta-analysis comparing mothers and fathers’ levels of sensitive parenting demonstrated that mothers indeed presented higher levels of sensitivity than fathers, but that the small-magnitude effect size was significantly weaker than anticipated based on evolutionary theories of parenting (Deneault et al., 2022a). In the case of activation parenting, the evidence thus far is mixed, with some studies finding gender differences in activation parenting (e.g., Schoppe-Sullivan et al., 2013) and others not (e.g., Olofson & Schoppe-Sullivan, 2022). The current study seeks to synthesize the available literature using a systematic review and meta-analysis to examine whether fathers present higher levels of activation parenting than mothers in partnered parents, as would be expected based on activation relationship theory. Additionally, we examine if activation parenting levels are correlated between partnered parents to understand potential interparental influences in activation parenting. For both meta-analyses, study (e.g., year of publication, peer-reviewed status), sample (e.g., children’s sex, parents’ age, ethnicity), and measurement characteristics (e.g., type of measure, specific construct assessed) are tested as potential moderators.

### 1.1 | Activation parenting

Traditional parenting research, which reflected the childrearing division of labor in traditional households, revolves around a model in which fathers were the providers for the family, with mothers being responsible for childcare (see Cabrera et al., 2000). Some models of parenting, rooted in evolutionary theory, rely on this traditional view to describe gendered differences in parenting behaviors. One example of such a theory is activation relationship theory, which proposes that parents may occupy different relational functions within the parent-child dyad, with mothers more often engaging in nurturing and sensitivity-oriented interactions, and fathers more often engaging in activation-oriented interactions involving stimulation and risk regulation (Paquette, 2004a, 2004b; Paquette & Puentes-Neuman, 2024). Under this view, parent-child interactions characterized by caregiving involvement tend to emphasize responsiveness to the child’s basic needs (e.g., responding to signals of distress), whereas activation-oriented interactions emphasize stimulation and the child’s openness to the external world (Paquette & Bigras, 2010; Paquette & Puentes-Neuman, 2024). This latter process may be facilitated by different types of activation behaviors, such as stimulating behaviors toward the child, often taking the form of physical play (e.g., tickling, gently pushing, play-fighting; Paquette, 2004a). Activation behaviors also include a limit-setting component that ensures that the child is not overstimulated, or overactivated (e.g., preventing excessive aggressivity during parent-child physical play), nor underactivated, which could increase child’s anxiety (Feldman & Shaw, 2021; Paquette & Puentes-Neuman, 2024). Activation parenting behaviors are expected to promote children’s capacity to safely explore their environment and engage in healthy, controlled risk-taking.

A recent systematic review of parental activation synthesized different forms of behaviors that could be grouped under activation parenting (Feldman & Shaw, 2021). In the context of the current study, we follow Feldman and Shaw’s (2021) definition of activation parenting, which includes, in addition to activation parenting, similar and related parenting behaviors, namely challenging parental behaviors and physical/rough-and-tumble play. First, challenging parenting behaviors are closely related to activation parenting behaviors, without the emphasis on limit-setting. They include both physical play and socio-emotional components that aim to challenge the child to explore and act in ways beyond their comfort zone (Majdandžić et al., 2016). These types of behaviors are thought to be beneficial in promoting children’s assertiveness and capacity to overcome challenges (Majdandžić et al., 2016). Some studies report links between challenging parenting behaviors and positive outcomes in children (e.g., decreased anxiety in children; Lazarus et al., 2016; Majdandžić et al., 2018), while others do not find evidence for such outcomes (e.g., Deneault et al., 2022b). Second, physical/rough-and-tumble play represents the behavioral component of play behaviors “that appear aggressive, but are performed in a non-aggressive, playful manner” (Feldman & Shaw, 2021, p. 421; Pellegrini, 2002). Rough-and-tumble play includes play-oriented behaviors such as jumping, chasing, and wrestling (Pellegrini & Smith, 1998). According to St George & Freeman’s (2017) meta-analysis, father-child rough-and-tumble play is moderately correlated with higher social competence, emotional skills, and self-regulation.

### 1.2 | Differences in caregiving behaviors between fathers and mothers

Some scholars argue that parents may be more similar than different given the current landscape of family dynamics. Cabrera et al. (2000) explain that sociological factors such as mothers’ increased workforce participation in the Western world have brought forth a redefinition of parental responsibilities. This idea is supported by numbers showing that fathers are more involved than ever before in childrearing, with a 3-to-6-fold increase in time spent directly caring for children across a single generation (Bakermans-Kranenburg et al., 2019). As a result, parental roles may have shifted toward a more equalitarian division of childrearing labor (Schoppe-Sullivan & Fagan, 2020), even though mothers still tend to assume the majority of caregiving (Aviv et al., 2025). Contemporary parenting, for both mothers and fathers, would involve a more balanced combination of acting as a breadwinner and a caregiver for children (Harrington, 2022; McGill, 2014), making parents more similar than different with respect to their behaviors (Fagan et al., 2014).

In order to test the proposition that parents’ behaviors may be more similar than different, Deneault et al. (2022a) examined differences in maternal and paternal sensitivity in partnered parents. Across 93 studies (*N* = 10,980 child-father dyads; 11,291 child-mother dyads), they only found a small difference in favor of levels of maternal sensitivity compared to paternal sensitivity (*d* = .27). Importantly, in their moderator analysis, they found that the gap in sensitivity across parental gender had narrowed to the point of being non-significant in studies from the last decade compared to older studies. Relatedly, the difference was non-significant in European countries, which generally have a more equalitarian division of childrearing due to policy efforts in the European Union (Sigle-Rushton et al., 2013). Their results may suggest that biologically-driven differences in parental behaviors may not be as large as expected, and that parents are more similar than different in their levels of parental sensitivity, especially in recent years. However, Deneault et al.’s (2022a) meta-analysis focused on behaviors that are considered to be in the maternal domain, leaving an empirical gap in comparing behaviors that are considered in the paternal domain (e.g., activation parenting) across fathers and mothers.

### 1.3 | Activation parenting and gender differences

Our first research question aims to compare fathers’ and mothers’ levels of activation parenting. On a theoretical level, activation relationship theory would suggest gaps in activation parenting across parents, given that activation parenting is considered one of the primary functions of fathering, along with protecting and providing for his family (Paquette, 2004a; Paquette & Puentes-Neuman, 2024). Alternatively, we may expect parents to show more similar levels of activation parenting, consistent with recent findings on parental sensitivity (Deneault et al., 2022a) and studies finding no gender differences in activation parenting. However, despite the increase in paternal involvement in the last decades, mothers still bear the majority of caregiving tasks (Aviv et al., 2025; Martínez-Pastor et al., 2024). Given that some studies find that fathers may engage in more physical play than mothers (Paquette & Puentes-Neuman, 2024; Paquette et al., 2003), it could be that their increased time in caregiving is reflected in more time spent engaging in activation parenting. Additionally, there may be biological differences inherent to sex, including brain differences among mothers and fathers and evolutionary-driven gender role differences (Feldman, 2023; Möller et al., 2013). In such a case, differences may still be identifiable between parents.

The evidence within the scientific literature is currently mixed about differences in mothers’ and fathers’ levels of activation parenting. While the founding work studying physical play (e.g., Crawley & Sherrod, 1984; Dickson, 1994; Weston, 1982) and activation parenting (e.g., Paquette et al., 2003) report that fathers engage in more activation parenting, more recent research does not consistently support gendered differences in such behaviors, notably (but not only) when studying challenging parenting behaviors (e.g., Majdandžić et al., 2016; Zaman, 2012). These mixed findings may benefit from a systematic synthesis to compare the levels of activation parenting in mothers and fathers across multiple studies.

### 1.4 | Associations in activation parenting between parents

Our second research aim is to examine the correlation between partnered mothers’ and fathers’ levels of activation parenting. This question, while distinct from differences in levels of activation parenting across parents, can provide important information on associations between parental behaviors across gender within a family. As stipulated by Schwartz’s assortative mating theory (2013), individuals tend to affiliate themselves with people who are similar to them on various levels (e.g., socioeconomic, values, ethnicity, religion). On the one hand, coparents may influence one another in adopting parenting practices that are enjoyable for the child, therein sharing similar parenting practices (Cox & Paley, 2003). On the other hand, assortative mating may also suggest that parents share similar values, which could be more traditional gender role views, in which case parents may not show correlated levels of activation parenting. Given that agreement on parenting practices represents an important component of coparenting according to Feinberg’s (2003) theory of coparenting, a significant correlation between mothers and fathers’ activation parenting levels may be beneficial to the well-being of both children and coparents. Additionally, it is possible that parents may adjust similarly to their child’s temperamental disposition (e.g., the parents of a difficult child would both use less activation parenting behavior) or condition (e.g., autism, attention deficit and hyperactivity disorder), which may contribute to an association between a mother’s and a father’s activation behaviors. Furthermore, most studies that document the correlation between partnered parents’ activation parenting levels find moderate effect sizes (e.g., *r*= .37, Grossman & Grossman, 2000; *r* = .45, Lazarus et al., 2018; *r* = .38, Majdandžić et al., 2016). Alternatively, some empirical evidence suggests a weak, sometimes null correlation (e.g., *r* = .20, Ghosh, 2024; *r* = .08, Majdandžić et al., 2014; *r* = .06, Olofson & Schoppe-Sullivan, 2022), which calls for meta-analytical work in order to synthesize the available empirical evidence.

### 1.5 | Current study

#### 1.5.1 | Research questions and hypotheses

Considering the current state of knowledge on genderbased differences and associations in parenting behaviors and the lack of convergence on empirical differences in activation parenting, the current study aims to synthesize the literature to inform differences and associations in activation parenting based on parental gender. First, we ask (1) do mothers and fathers exhibit similar levels of activation parenting? Despite the theoretical assumption that fathers tend to engage in more activation parenting, the mixed empirical evidence prevents us from making a clear hypothesis. The contemporary shifts in both parenting roles and division of labor (see Cabrera et al., 2000; Van der Gaag et al., 2023), along with the potential specialization of gendered parenting behaviors (Möller et al., 2013; Paquette & Puentes-Neuman, 2024; Paquette et al., 2004a), further preclude us from hypothesizing the directionality of the present meta-analysis. Second, we ask (2) are partnered parents’ activation parenting behaviors correlated? Based on the associative mating theory, and on past studies that found moderate correlations in partnered coparent’s behaviors (e.g., Deneault et al., 2022a), we hypothesize that mothers’ and fathers’ activation parenting levels will be correlated, with a moderate effect size. The present study may enhance the understanding of gender differences and similarities in parenting practices. This knowledge is important to tailor interventions that effectively meet the needs of both mothers and fathers, particularly as fathers tend to be less likely to engage in these programs (Panter-Brick et al., 2014).

To answer these research questions, we rely on a systematic review and meta-analyses. The systematic review process enables the identification of all relevant studies that compare mothers’ and fathers’ levels of activation parenting, while the meta-analyses allow for a synthesis of the results to estimate effect sizes of differences and associations. This methodology also allows for the testing of multiple possible moderators, which may account for heterogeneity in activation parenting studies and could provide a deeper and more nuanced understanding of gender differences in activation parenting.

#### 1.5.2 | Potential moderators

For both meta-analyses, we considered study-level, sample-level, and measurement-level characteristics as potential moderators. With respect to study characteristics, we examined the publication year of studies as a proxy for the year of data collection, given that this information is rarely reported in studies. Parenting roles have evolved in recent decades (Cabrera et al., 2000, 2014; Van der Gaag et al., 2023), and while mothers continue to bear most caregiving responsibilities, father involvement has significantly increased over the years. Thus, we tested whether differences and correlations in activation parenting change over time. We also considered the type of publication (i.e., peer-reviewed vs. not peer-reviewed) as a moderator, given that peer-reviewed publications may be more likely to fall prey to publication bias in favor of significant effect sizes. Relatedly, we examined if the overall quality of studies moderated the magnitude of effect sizes.

In terms of sample characteristics, we examined potential differences due to parents’ ethnocultural characteristics and socioeconomic status (SES). Most activation parenting research (and, to some extent, parenting research broadly), has been conducted in white parents from a mid-high SES background. While many studies did not find differences in activation parenting based on these characteristics, others have reported a significant moderating role of education or ethnic origin on parenting behaviors such as verbal interactions or challenging parental behaviors (Cabrera et al., 2011; Deneault et al., 2022b). We also considered the potential effect of child sex and age. According to Feldman and Shaw’s systematic review (2021), paternal activation parenting could increase in intensity with children’s growth, but the link between the quantity of activation behaviors and child age remains unclear due to mixed empirical evidence. Concerning child sex, while Paquette’s (2004a) activation relationship theory supposes that parents may engage in more activation with boys, most studies do not find a significant difference between fathers’ activation behaviors toward boys and girls (Feldman & Shaw, 2021).

In terms of measurement characteristics, we examined for potential differences based on the type of activation parenting behavior assessed (i.e., activating parenting behaviors, challenging behaviors, physical/rough-and-tumble play). Despite their conceptual similarity (Feldman & Shaw, 2021), there are differences between these types of activation behaviors and their measurement, which may influence the magnitude of differences and correlations. We also considered assessment methods, as studies report divergent findings when using self-report or observational methods, even when measuring the same construct (e.g., Majdandžić et al., 2016). We also considered the psychometric properties of instruments as they can vary greatly across studies, and influence the quality of results.

## 2 | METHODS

This systematic review was conducted following the guidelines of the Preferred Reporting Items for Systematic Reviews and Meta-Analyses (PRISMA) framework (Page et al., 2021). The protocol was preregistered on OSF: https://osf.io/4tcgx/?view_only=ee01f3ff5b514ecf9b56523d189d8df7. The study followed the preregistered analysis plan, with one deviation. Specifically, parental playfulness was excluded from the final analyses following peer review feedback that raised concerns about its conceptual fit with the study’s primary research questions.

### 2.1 | Search strategy

**FIGURE 1:**
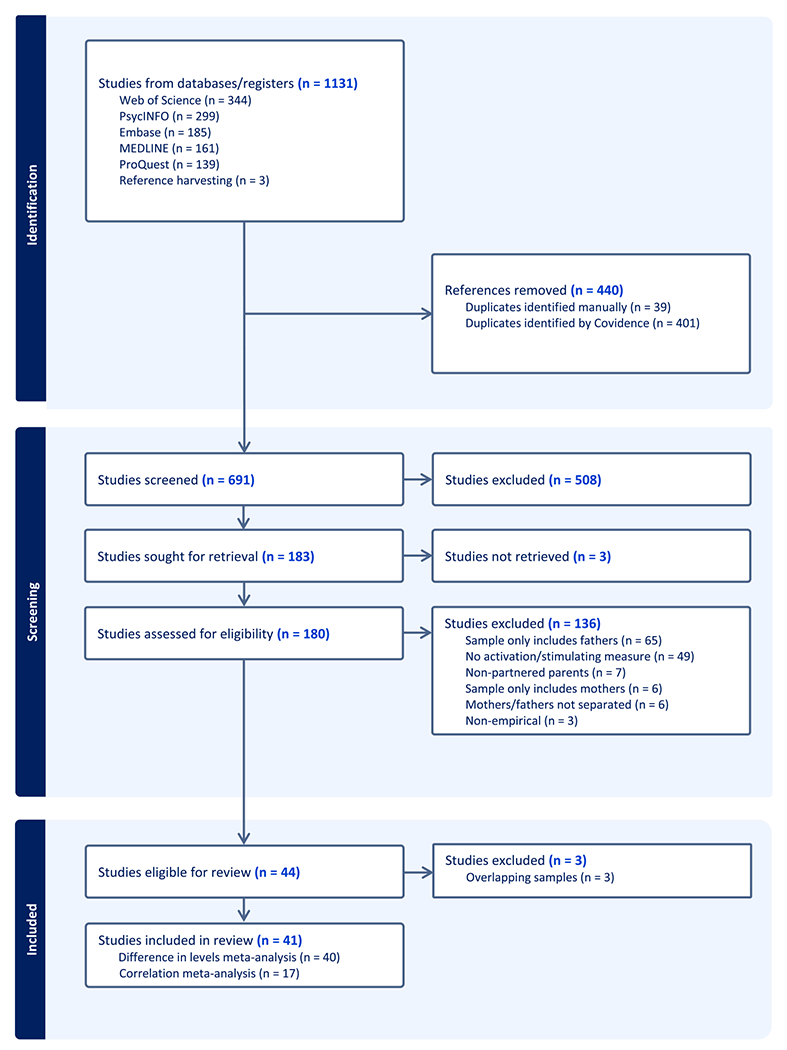
PRISMA flowchart of study selection.

The search strategy used a mixed method. First, an automated search was conducted across five databases (EMBASE, PsycINFO, MEDLINE, ProQuest Dissertations & Theses, and Web of Science). The search was first conducted on May 1, 2024, and last updated on January 1, 2025. The database search revolved around two core concepts: (1) activation parenting and synonymous or related terminology (e.g., rough-and-tumble play, challenging parenting behavior, physical play) and (2) fathers (e.g., paternal, father, parent). The two core concepts were linked with the Boolean operator “AND,” while the different terms within core concepts were linked using “OR.” Second, we conducted a manual reference harvesting using a backward search in the reference list of selected articles to identify additional studies. Our mixed search identified 691 non-duplicate records to screen (see Figure 1 for PRISMA flowchart).

### 2.2 | Study selection

The eligibility of records was determined based on the following selection criteria: (1) using empirical data (excluding case studies with *N* < 5 families), (2) including partnered mothers and fathers, (3) measuring activation parenting (or a related construct included in our search strategy) separately for mothers and fathers, and (4) providing a statistical effect size that could be used for at least one of the meta-analyses. The systematic review management software Covidence (Veritas Health Innovation, 2020) was used to assess the eligibility of records. A team of five coders screened abstracts and then full texts, with each record being screened by two independent coders. The inter-rater agreement was 88.0% for abstract screening and 86.7% for full-text screening. Disagreements were resolved by the first author in consultation with the last author. Following these study selection steps, 44 articles remained eligible for inclusion.

### 2.3 | Data extraction and quality assessment

Coders extracted study characteristics, sample characteristics, measurement characteristics, and effect sizes using a standardized extraction form in Covidence. Study characteristics extracted included (a) publication year and (b) the type of publication (i.e., article, book chapter, thesis/dissertation). Sample characteristics included (a) continent of data collection, (b) socioeconomic status (low, mixed, or mid-high), (c) parents’ racial background (% white), (d) children’s age (in months), and (e) children’s sex. Measurement characteristics included (a) the type of behavior assessed (i.e., activation parenting, challenging behaviors, or physical/rough-and-tumble play) and (b) the assessment method (i.e., observation or questionnaire), and (c) the psychometric properties of instruments (e.g., intra-class correlation [ICC] for observational measures, Cronbach’s alpha for questionnaires). Effect sizes pertaining to the levels of activation were extracted as well as correlations between levels of activation in mothers and fathers. Two independent coders extracted data from every article, and disagreements were resolved by the first author. Inter-rater reliability was high for categorical (> 90%) and continuous variables (ICC > .85).

Study quality was evaluated by the same five coders who conducted data extraction using an adapted version of the National Heart, Lung, and Blood Institute (NIH; n.d.) Quality Assessment Tool for Observational Cohort and Cross-Sectional Studies (see Supplemental Table 1). Quality assessment criteria related to the recruitment strategy, sociodemographic description, psychometric properties of measures, and overall quality. Inter-rater agreement was good (> 80%). Disagreements were resolved by the first author. For the purpose of moderator testing, we calculated a percentage quality score based on the number of high-quality items out of the number of applicable items for each article. Higher scores indicate higher quality.

### 2.4 | Data synthesis and analysis

Among the 44 studies eligible for inclusion, some reported on data from overlapping samples. Overlaps were rigorously examined to ensure that studies reported on different data (e.g., different time points, different scales). When data were the same across studies, we selected the one with the larger *N* and more readily extractable data (see Supplemental Table 2 for a description of selection decisions). After accounting for overlaps, 41 studies were included in our meta-analyses.

We synthesized effect sizes for the first meta-analysis (difference between mothers and fathers) as standardized mean differences (Hedge’s *g*). Studies reporting different types of effect sizes (e.g., *p*-values, *t*-test) were converted into standardized mean differences prior to analyses. For the second meta-analysis (correlation between parents), we synthesized data as Pearson’s correlations (*r*). Data were transformed into Fisher’s *z* for the analysis, given that variances vary based on the magnitude of correlations (Borenstein & Hedges, 2019). The results were converted back to correlations for ease of interpretation. For both meta-analyses, consistent with guidelines by Rosenthal (1995), a non-significant difference without specific effect sizes was assigned a *p*-value of .50. We conducted the meta-analyses by including all eligible studies into a global activation parenting category comprising physical play, activation parenting, and rough-and-tumble-play.

Data were analyzed using multilevel random effects meta-analyses. The multilevel approach allows for the inclusion of dependent effect sizes (e.g., data from multiple time points) while accounting for the dependency between these effects. Consistent with current guidelines for the synthesis of dependent effect sizes (Harrer et al., 2021), we conducted a multilevel meta-analysis with the correlated and hierarchical effects (CHE) model, which allows for the inclusion of complex data structures (i.e., correlated *and* hierarchical effect sizes) (Pustejovsky & Tipton, 2022). We estimated robust confidence intervals and *p*-values with the robust variance estimation (RVE) with a small-sample adjustment (Tipton & Pustejovsky, 2015). We interpreted the magnitude of effect sizes with calibrated guidelines for psychological research (Funder & Ozer, 2019). For the first meta-analysis, we considered Hedge’s *g* of approximately .20, .41, and .63 to be small, moderate, and large in magnitude, respectively. For the second meta-analysis, we considered Pearson’s *r* of .10, .20, and .30 to be small, moderate, and large, respectively.

We assessed the presence of publication bias by examining funnel plots and conducting Egger’s test and a *p*-curve analysis. We evaluated heterogeneity via the prediction interval (Borenstein, 2022). We also report the *Q* statistic and the *I^2^* value. We used meta-regressions to test for moderator variables. We evaluated significance for continuous moderators via the RVE *p*-value. For categorical moderators, the *Q* moderator value served as an omnibus test of significance. Significant moderators were probed with a pairwise Wald test. We conducted all analyses and graphics in R (R Core Team, 2024), using the packages *esc* (Lüdecke, 2019), *metafor* (Viechtbauer, 2010), *dmetar* (Harrer et al., 2019), *metasens* (Schwarzer et al., 2019), *clubSandwich* (Pustejovsky, 2020), *psych* (Revelle, 2026), and *ggplot2* (Wickham, 2016). The data and analysis code are available online: https://osf.io/4tcgx/?view_only=ee01f3ff5b514ecf9b56523d189d8df7

## 3 | RESULTS

### 3.1 | Meta-analysis 1: Differences in levels between partnered parents

#### 3.1.1 | Study and effect sizes characteristics

A summary of study characteristics can be found in Table 1. This meta-analysis included 59 effect sizes drawn from 40 studies (38 unique samples). We first report descriptive statistics at the study level (i.e., to avoid counting the same sample multiple times for characteristics that are stable within a study, such as the type of publication or continent of data collection). The studies included a total of 3607 unique fathers (range = 5 to 634, median = 53) and 3707 unique mothers (range = 5 to 660, median = 54) who were partnered together. Some samples included a few mothers who participated while their partner did not participate, explaining the difference in *N* across parents. Approximately half of the children were boys (53.2%). Across samples for which racial background was reported (*k*= 17), most parents were white (80.8% of fathers and 81.9% of mothers). Families in most samples were from a mid-high SES background (61.8%, *k* = 21), with the rest having mixed SES (35.3%, *k* = 12) or low SES (2.9%, *k* = 1). Samples were collected in North America (76.3%, *k* = 29), Europe (10.5%, *k* = 4), Asia (5.3%, *k* = 2), Oceania (5.3%, *k* = 2), or multiple continents (2.6%, *k*= 1). Most studies were published in peer-reviewed articles (63.2%, *k* = 24), with the rest being theses/dissertations (34.2%, *k*= 13) or book chapters (2.6%, *k* = 1). Studies were published between 1977 and 2024. The mean quality score of studies was 58%, with a range of 25% to 85% (see Supplemental Table 3).

We then report descriptive statistics at the effect size level (i.e., to adequately report characteristics that may vary *within* a study, such as child age, which would vary at each time point in a longitudinal study). Fathers were on average 34.3 years old (range of means: 26.0 to 39.7), and mothers were on average 31.7 years old (range of means: 23.6 to 36.9) at the assessment of their parenting behaviors. Children were, on average, 41.4 months old (range: 3 months to 238.7 months old [19.9 years]; median: 35 months). Most effect sizes assessed parents’ rough-and-tumble/physical play (62.7%, 37 effect sizes), followed by challenging behaviors (32.2%, 19 effect sizes), and activation parenting (5.1%, 3 effect sizes). Most of the effect sizes were measured using observation (71.2%, 42 effect sizes), while the rest were measured using questionnaires (27.1%, 16 effect sizes) or interviews (1.7%, 1 effect size).

#### 3.1.2 | Meta-analysis 1 results

**FIGURE 2:**
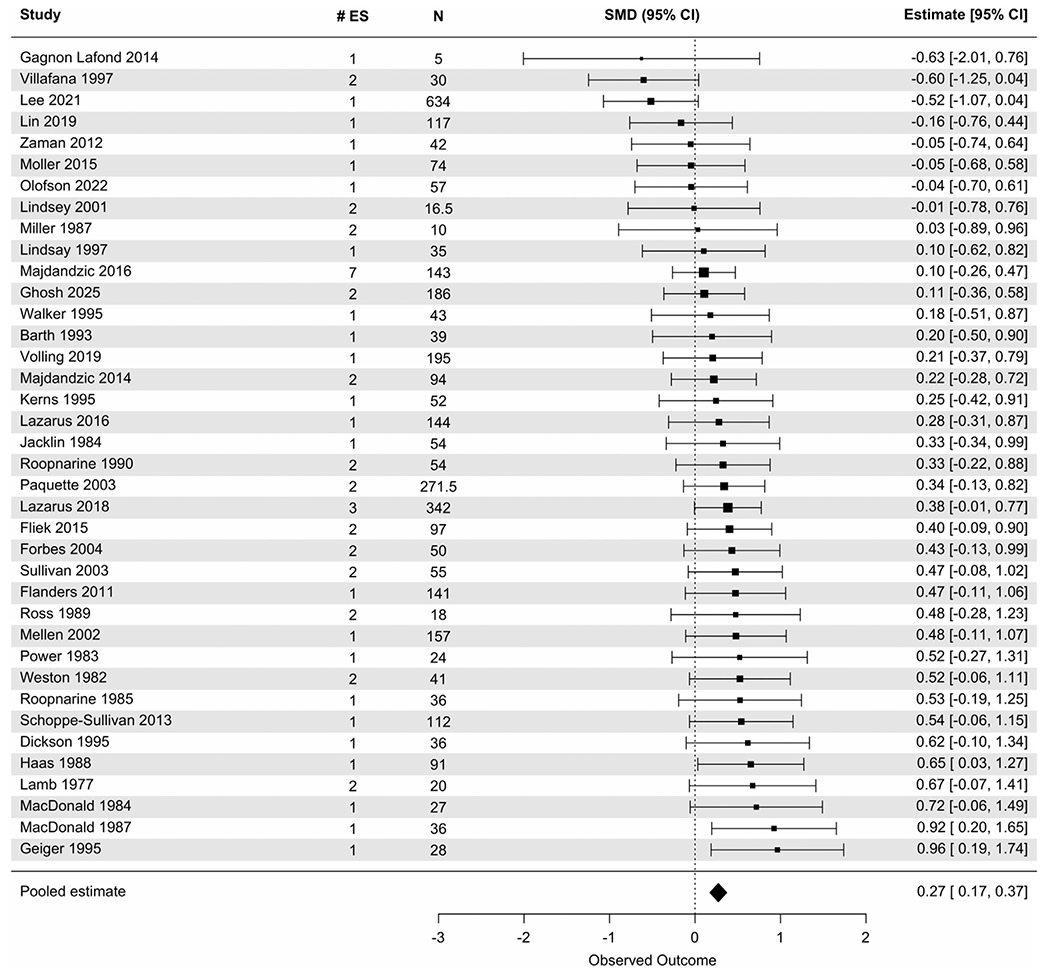
Forest plot of the difference between fathers’ and mothers’ activation parenting levels.

The meta-analysis revealed that fathers presented significantly higher levels of activation parenting than mothers, with a synthesized effect size of a small magnitude: *g* = .27, 95% CI [.17, .37], *p* < .001 (see Figure 2). Results were consistent across different levels of Rho; for ease of reporting, results for Rho = .80 are reported below. There was no evidence of publication bias based on the funnel plot (see Supplemental Figure 1) nor Egger’s test (*t* = .71, *p* = .49). We did not find evidence of selective reporting using the *p*-curve analysis. Indeed, 81.4% of effect sizes were significant at the*p* < .25 level, and the test of right skewness was significant (zHalf = −47.24, *p* < .001), while the test of flatness was not significant (zHalf = 47.54, *p* = .99). Sensitivity analyses for the *p*-curve analysis using the aggregate effect sizes revealed similar results.

With respect to heterogeneity, the *Q* test was significant (*Q* = 719,22, *p* < .001), and the prediction interval suggested the presence of heterogeneity, with values ranging from a small negative difference to a very large positive difference (−.28, .83). The between-sample and between-effect sizes *I^2^* were .01 and .06, respectively. Moderation analyses (see Table 2) revealed that the gap between fathers’ and mothers’ global activation parenting levels decreased as a function of publication year, which was used as a proxy for data collection year (*b* = −.16, *p* = .002; see Supplemental Figure 2 for a graphical depiction of the effect). Additionally, the type of activation behavior assessed emerged as a significant moderator (*Q_M_* = 6.39, *p* = .01). The difference in activation levels across parents was moderate in magnitude for physical/rough-and-tumble play (*g* = .39, 95% CI [.27, .51]) and small in magnitude for challenging parenting (*g* = .16, 95% CI [.02, .31]); the activation parenting category could not be included as it had too few effect sizes. Given that challenging behaviors only started being assessed in the last decade, it is possible that the moderation effect on publication year is due to an increased number of studies examining challenging behaviors compared to before (see Supplemental Figure 3 for a graphical depiction of the moderation by year for each type of behavior). We modeled the publication year moderation effect for each type of behavior. The publication year effect was not significant for challenging behaviors (*g* = .004, 95%. CI [−.114, .122], *p* = .92), nor physical/rough-and-tumble play (*g* = −.074, 95% CI [−.203, .056], *p* = .23), suggesting that the moderator effect was an artifact of the type of behaviors assessed changing over time. No other significant moderators were identified (child age and sex, ethnic background, publication year, quality assessment, questionnaire reliability, behavior assessment method, and publication type). The continent of data collection could not be tested due to continents other than North America having too few degrees of freedom (i.e., < 4.0; Tanner-Smith et al., 2016). The inter-rater reliability of observational measures was not tested, given that all studies met the “good” reliability threshold (e.g., ICC ≥ .70; Hallgren, 2012).

### 3.2 | Meta-analysis 2: Correlations between partnered parents

#### 3.2.1 | Study and effect sizes characteristics

A summary of study characteristics can be found in Table 1. This meta-analysis comprised 28 effect sizes drawn from 17 studies (14 unique samples). Regarding study-level descriptive statistics, the samples included a total of 2231 unique families, with a range for individual studies of 30 to 634 families (median = 106 families). Approximately half of the children were boys (54.6%). Across samples for which racial background was reported (*k* = 9), most parents were white (72.6% of fathers and 72.2% of mothers). Families in most samples were from a mid-high SES background (71.4%, *k* = 10), with the rest having mixed SES (21.4%, *k* = 3) or low SES (7.1%, *k* = 1). Samples were collected in North America (57.1%, *k* = 8), Europe (28.6%, *k* = 4), Oceania (7.1%, *k* = 1), and Asia (7.1%, *k* = 1). Most studies were published in peer-reviewed articles (71.4%, *k* = 10), with the rest being theses/dissertations (21.4%, *k* = 3) or book chapters (7.1%, *k* = 1). Studies were published between 1997 and 2024. The mean quality score of studies was 62%, with a range of 42% to 83% (see Supplemental Table 3).

Regarding effect size-level descriptive statistics, fathers were on average 35.7 years old (range of means = 26.0 to 39.7) and mothers were on average 33.2 years old (range of means = 23.6 to 37.0) at the assessment of their parenting behaviors. Children were, on average, 58.3 months old (range = 4.14 months to 238.7 months old [19.89 years]; median = 45 months). Most effect sizes assessed parents’ challenging behaviors (64.3%, 18 effect sizes), followed by rough-and-tumble/physical play (28.6%, 8 effect sizes), and activation parenting (7.1%, 2 effect sizes). Approximately half of the effect sizes were measured using observation (42.9%, 12 effect sizes), while the rest were measured using questionnaires (57.1%, 16 effect sizes).

#### 3.2.2 | Meta-analysis 2 results

**FIGURE 3:**
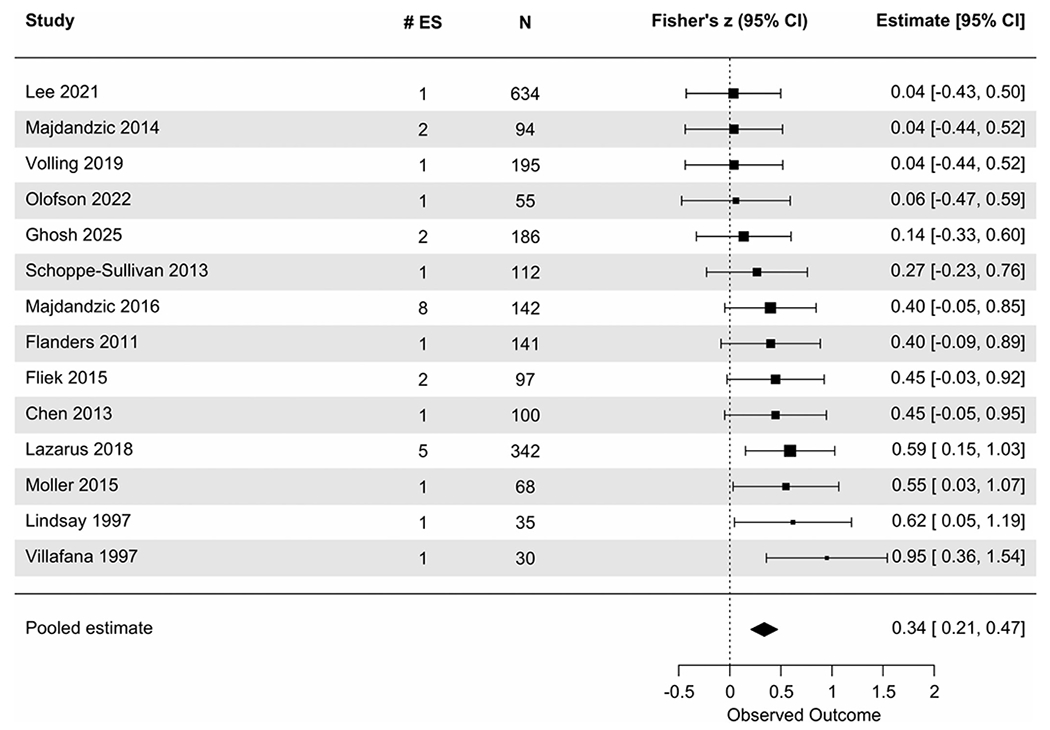
Forest plot of the correlation between fathers’ and mothers’ activation parenting levels.

The meta-analysis revealed a significant association of a large magnitude between global activation parenting behaviors across partnered parents: *r* = .33, 95% CI [.19, .45], *p* < .001 (see Figure 3). Results were consistent across different levels of Rho. For ease of reporting, the results for Rho = 0.80 are reported below. There was no evidence of publication bias based on the funnel plot (see Supplemental Figure 4) nor Egger’s test (*t* = .51, *p* = .63). We did not find evidence of selective reporting using the *p*-curve analysis. Indeed, 97% of effect sizes were significant at the*p* < .25 level, and the test of right skewness was significant (zHalf = −38.90, *p* < .001), while the test of flatness was not significant (zHalf = 40.12, *p* = .99). Sensitivity analyses for the *p*-curve analysis using the aggregate effect sizes revealed similar results.

With respect to heterogeneity, the *Q* test was significant (*Q* = 368.10, *p* < .001), and the prediction interval suggested the presence of heterogeneity, with values ranging from a small negative correlation to a very large positive correlation (−.13, .67). The between-sample and between-effect sizes *I^2^* were .04 and .01, respectively. Moderation analyses (see Table 3) revealed a significant moderation effect of publication year (*b* = −.13; see Supplemental Figure 2 for a graphical depiction of the effect). Other moderators (i.e., type of behavior, type of assessment, child age and sex, ethnic background, quality assessment, and questionnaire reliability) were not significant moderators. Location of data collection and peer-reviewed status could not be tested, as they had too few degrees of freedom to be tested. The inter-rater reliability of observational measures was not tested, given that all studies met the “good” reliability threshold (e.g., ICC ≥ .70; Hallgren, 2012).

## 4 | DISCUSSION

The goal of the present study was to (1) examine the comparative levels of activation parenting exhibited by fathers and mothers in partnered parents, and (2) determine whether partnered parents’ activation parenting behaviors are correlated. The use of meta-analyses to answer these questions allowed for a global synthesis of the current mixed literature on activation parenting, and to test the role of multiple potential moderating variables. The meta-analyses revealed a significant, but small, difference in activation parenting in favor of fathers, and a large correlation between partnered parents’ activation parenting. Our results contribute to the parenting literature by deepening our understanding of activation parenting, a less studied parental behavior, and gender differences and similarities in parenting behaviors. Furthermore, the present findings can help design parenting interventions that foster activation parenting and benefit both mothers and fathers, as well as their children.

### 4.1 | Fathers show slightly higher levels of activation parenting

Our first meta-analysis found that fathers tend to engage in more parental activation behaviors than mothers, but only to a small extent (*g* = .27). Thus, although there is a statistical difference, it remains modest and does not suggest a rigid differentiation between gendered parental behaviors, at least not concerning activation parenting. From a theoretical perspective, parental activation is described as a predominantly paternal function according to the activation relationship theory (Paquette, 2004a, 2004b; Paquette & Puentes-Neuman, 2024). This theory postulates that fathers primarily act as stimulators and facilitators of exploration and risk-taking in children, while mothers tend to play a more caregiving and sensitive role. However, the results of the present meta-analysis, together with those of Deneault et al. (2022a) showing mothers’ only slightly higher levels of parental sensitivity, nuance this vision by demonstrating that the gap in parental behaviors among genders is relatively small. Overall, there seems to be a tendency for fathers to engage in more activation and mothers to engage in more sensitive behaviors, but fathers and mothers alike engage in both types of behaviors.

Furthermore, the meta-analysis revealed that the gap between mothers’ and fathers’ levels of overall parental activation has decreased over time. This may be a result of changes in parental roles in contemporary families, with increased levels of direct paternal involvement in childcare (Bakermans-Kranenburg et al., 2019). While these results align with the findings of Deneault et al.’s (2022a) metaanalysis showing a decrease in gender differences toward parental sensitivity over time, further analyses revealed an alternative explanation. Specifically, further moderator analysis revealed a significant difference between the types of activation parenting. Following the publication of Paquette’s (2004a) seminal work on the activation relationship, new aspects of activation parenting started being examined, such as challenging behaviors. As a result, we sought to determine whether the publication year moderation could be an artifact of the type of behavior assessed. It appears that trends in the types of behaviors that researchers choose to assess may indeed contribute to the significant publication year moderator effect. Notably, research on physical play has seemingly stalled in the recent decade, compared with an increase in studies on challenging behaviors (see Supplemental Figure 3). In turn, this limits our ability to detect whether publication year effects are present for each type of activation parenting (e.g., too few recent studies on physical play to adequately test if differences are now smaller compared to before). In order to answer this question, future studies should assess different types of activation parenting.

These studies could also help understand why differences emerged across types of activation parenting. Specifically, the gender difference in activation parenting levels was moderate for physical play, but small for challenging parenting. The conceptual distinctions between such constructs could explain these results. As explained in Feldman and Shaw’s (2021) narrative review, physical play could represent a purely behavioral, play-oriented construct, while challenging parenting behaviors include behavioral and verbal components. Thus, our results suggest that while mothers and fathers are only slightly distinct when it comes to activation parenting, fathers may be more prone to using behavioral strategies (such as physical play) with their children (see Paquette & Puentes-Neuman, 2024). Future research could shed light on potential gender differences based on the strategies used to activate the child. Additionally, future research is needed to understand whether this gap of a small magnitude between mothers’ and fathers’ activation behaviors could be compounded over time, such that receiving a bit more activation from fathers over the course of an entire childhood could lead to different outcomes for children.

### 4.2 | Activating father, activating mother

The result of the second meta-analysis shows a strong correlation between mothers’ and fathers’ levels of parental activation (*r* = .33). It is possible that parents who have a tendency to engage in more challenging behaviors, whether with children or not, are more likely to choose one another as partners, leading to more similar parenting practices (Schwartz, 2013). Given that activation parenting is usually an enjoyable play activity with the child, it is possible that when a parent observes their partner fostering the conditions for a fun, challenging interaction with their child, they themselves adopt similar behaviors to foster enjoyable interactions when they play with their child (Cox & Paley, 2003). While not tested in the current study, it is also possible that both parents adjust their parental behavior to their child’s temperamental needs for stimulation and physicality in play in similar ways (Majdandžić et al., 2018), which could explain why parents use similar levels of activation parenting. Further research is needed to test this potential explanation.

These results are consistent with the meta-analysis on observed parental sensitivity, which found a moderate correlation between partnered mothers and fathers’ sensitivity levels (*r* = .23; Deneault et al., 2022a). More research is needed to understand if similarity in multiple types of parental behaviors could foster coparenting agreement and, in turn, promote well-being within the family system (Feinberg, 2003). Additionally, more research is needed to examine determinants of activation parenting behaviors, a topic comparatively less examined than the determinants of parental sensitivity.

### 4.3 | Practical implications

The current study suggests that both fathers and mothers engage in activation parenting behaviors, with fathers exhibiting slightly higher levels of general activation parenting. Since activation parenting may be beneficial for children (Feldman & Shaw, 2021), it may be relevant to encourage parents, regardless of gender, to incorporate activation parenting behaviors in their parenting, while remaining sensitive to the children’s needs and limits. More work is needed on activation parenting interventions, but existing research suggests that there may be benefits for children’s assertiveness and capacity to overcome challenges (Majdandžić et al., 2016, 2018). Activation parenting has been shown to be strongly correlated with parental sensitivity, which reinforces its importance as a clinical focus (Bissonnette, 2019).

Furthermore, even if a shift toward a more equalitarian division of childrearing labor is apparent in contemporary parental roles (Schoppe-Sullivan & Fagan, 2020), mothers still tend to assume the majority of caregiving tasks and spend more time fulfilling childrearing responsibilities (Aviv et al., 2025). These trends highlight the importance of fostering programs and interventions in which fathers can recognize and identify themselves, notably by incorporating activating behaviors. Moreover, the results suggest that mothers could also be involved in such programs, as they also engage in these behaviors. A thorough body of literature reports that fathers are less inclined than mothers to engage in parenting programs (Fabiano, 2007; Panter-Brick et al., 2014). Therefore, it appears particularly important to invest in interventions that actively seek to include fathers and promote their involvement in caregiving. Empirical evidence suggests that when fathers do engage positively in childrearing, they can have a unique and lasting impact on their child’s development and well-being (Feldman, 2023; Pancsofar et al., 2010).

### 4.4 | Limitations

This study presents several limitations. First, the current body of literature on activation parenting is somewhat homogeneous when it comes to sample demographics. In fact, the vast majority of included studies rely on white, Western, and highly educated samples. These characteristics limit the external validity of our results, as they primarily reflect activation parenting practices among high SES parents. As such, this limited demographic representation should be considered when interpreting our results, given that race/ethnicity and SES are linked to activation parenting practices (Cabrera et al., 2011; Deneault et al., 2022b). However, to maximize the interpretability of our results, racial background was tested as a moderator in both meta-analyses but did not emerge as a significant moderating variable. Furthermore, activation parenting literature is somewhat limited and includes a wide range of different measures and constructs (e.g., physical play, challenging parenting behaviors), which could reduce the internal validity of the present study. These various types of activation parenting and their varied assessment methods may feature conceptual divergences, which remain understudied. This conceptual variety should be considered when interpreting the present results, as moderating analyses reveal slight differences in results when considering the specific constructs assessed. These limitations are inherent to included studies and seem inevitable when conducting systematic reviews and meta-analytical work. We sought to minimize the impact of these methodological limitations by conducting moderating analyses. However, further research could significantly contribute to the scientific literature by expanding parenting studies to more diverse samples and by considering how different types of activation parenting behaviors are different or similar, and whether they are associated with the same outcomes for child development.

### 4.5 | Conclusion

The present study shows that fathers engage in more activation parenting behaviors with their child than mothers, but that the difference is only small in magnitude. Our results showcase the importance of examining parental behaviors regardless of gender, given that both parents can activate children, even fathers may engage in more activation parenting. Additionally, activation levels were correlated among parents. This study emphasizes the need to examine parenting constructs across genders, given that mothers and fathers alike can exhibit a wide range of behaviors that may be beneficial for child development.

## Supplementary Material

Supplemental Material

Additional supporting information can be found online in the Supporting Information section at the end of this article.

## Figures and Tables

**TABLE 1:** Study characteristics.

Meta-analysis^d^	Study	Study type^e^	Continent^f^	Sample size		White (%)^g^	SES	Child		Activation parenting	
				Fathers	Mothers			Sex (% boys)	Age	Type	Method
1	Barth 1993	1	4	39	43		Mixed	48.9	63.5	Phys	Observation
2	Chen 2013	1	1	100	100		Mixed	60	103	Phys	Questionnaire
1–2	Deneault et al., 2022a ^a^	1	4	186	186	9	Mixed	49	9	CPB	Observation
1	Dickson, 1994	2	4	36	36		Mixed	52.8	12	Phys	Observation
1–2	Flanders, 2008	2	4	141	192		Mid-high	50	99	Phys	Questionnaire
1–2	Fliek, 2015	1	2	97	105		Mixed	58.1	51.2	CPB, Phys	Questionnaire
1	Forbes, 2004	1	4	50	50	86	Mixed	46	3, 6	Physical Play	Observation
1	Gagnon Lafond, 2014	2	4	5	5			20	48	Activation	Observation
1	Geiger, 1995	2	4	28	28		Mixed	50	13.2	Phys	Observation
1–2	Ghosh, 2024 ^a^	2	4	106, 135	106, 135	8	Mixed	49	18	CPB, Playfulness	Observation
1	Haas, 1988	2	4, 5	91	91		Mixed	48.3791	37	Phys	Questionnaire
1	Jacklin, 1984	1	4	54	54			55.6	45	Phys	Observation
1	Kerns, 1995	1	4	52	54	96.3	Mid-high	50	44.4	Phys	Observation
1	Lamb, 1977	1	4	20	20	100	Mixed	50	7.5, 12.5	Phys	Observation
1–2	Lazarus, 2016 ^b^	1	3	144	164	78	Mid-high	43.9	47.6	CPB	Questionnaire
1–2	Lazarus, 2018	1	3	342	342			21.4	238.7	CPB	Questionnaire
1–2	Lee, 2021	1	4	634	660	24.9	Low	44.85	43	Activation	Observation
1	Lin, 2019	1	1	117	121		Mid-high	58.1	30.4	Phys	Questionnaire
1–2	Lindsey, 1997	1	4	35	35	80	Mid-high	48.6	60.8	Phys	Observation
1	Lindsey, 2001	1	4	15, 18	15, 18	87.8	Mid-high	54.5	60.4	Phys	Observation
1	MacDonald, 1984	1	4	27	27	100	Mid-high	48.1	47.5	Phys	Observation
1	MacDonald, 1987	1	4	36	36			100	48	Phys	Observation
1–2	Majdandžić, 2014	1	2	94	94	95	Mid-high	56	30.8, 51.7	CPB	Observation
1–2	Majdandžić 2016 ^c^	1	2	128	127		Mid-high	45	4.1, 30.5, 12.6	CPB	Questionnaire
1–2	Majdandžić 2018 ^b,c^	1	2	142	144		Mid-high	43.8	52.2	CPB, Phys	Questionnaire
1	Mellen, 2002	2	4	157	157	85.4	Mixed		42	Phys	Questionnaire
1	Miller, 1987	2	4	12, 8	12, 8		Mid-high	0, 100	8.4	Phys	Observation
1–2	Möller, 2015	1	2	74, 68	76, 68		Mid-high	49	11.9	CPB	Questionnaire
1–2	Olofson & Schoppe-Sullivan, 2022	1	4	57	55	87	Mid-high	64.5	16.4	CPB	Observation
1	Paquette, 2003	1	4	75, 468	43, 468		Mixed, Low	50.7, 50.2	45.8, 37.5	Phys	Observation
1	Power, 1983	1	4	24	24	95.8	Mid-high	50	7.67	Phys	Observation
1	Roopnarine, 1985	1	4	36	36		Mixed	52.77	54	Phys	Observation
1	Roopnarine, 1990	1	1	54	54		Mid-high	50	12	Phys	Observation
1	Ross, 1989	1	4	18	18		Mid-high	100	35	Phys	Observation
1–2	Schoppe-Sullivan et al., 2013	1	4	112	112		Mid-high	51.8	49.4	Phys	Questionnaire
1	Sullivan, 2003	2	4	55	55	90.9	Mid-high	41.8	41.9	Phys	Observation
1–2	Villafana, 1997	2	4	30	30	100	Mid-high	100	13.1	Phys	Observation
1–2	Volling, 2019	3	4	195	195	85	Mid-high	57.44	12.5	Activation	Observation
1	Walker, 1995	2	4	43	43		Mixed	53.5	12	Phys	Observation
1	Weston, 1982	2	4	41	41		Mid-high	46.3	6.4	Physical Play	Observation
1	Zaman, 2012	2	4	42	42	73.8	Mid-high	50	54	CPB	Observation

*Note*: Superscript letters (a, b, c) identify groups of studies that share overlapping samples. Studies within each group were derived from the same sample and were treated accordingly in the meta-analyses.

d 1 = Meta-analysis comparing levels of paternal and maternal activation parenting; 2 = Meta-analysis examining the association between paternal and maternal activation parenting.

e 1 = Peer-reviewed article, 2 = Thesis, 3 = Book chapter.

f 1 = Asia, 2 = Europe, 3 = Oceania, 4 = North America, 5 = South America.

g Fathers’ ethnicity presented, based on the best available information in the study.

CPB, Challenging behaviors; Phys, Physical play.

**TABLE 2:** Moderator analysis for the difference in activation levels between parents.

Categorical moderators	*k*	*df*	*g*	95% CI	*Q_M_*	*p*
**Behavior type** ^a^					6.39	.01
Global activation	3	1.2	−.20	[−.99, .98]		
Challenging	19	5.8	.16*	[.02, .30]		
RTP	37	23.0	.37***	[.26, .47]		
**Behavior assessment method**					.47	.49
Observation	42	23.1	.25***	[.12, .36]		
Questionnaire	16	7.2	.31**	[.17, .44]		
**Continent of data collection** ^b^					–	–
Asia	3	1.0	.10	[−.99, .99]		
Europe	12	2.7	.18	[−.10, .42]		
North America	39	25.0	.28***	[.15, .40]		
Oceania	4	1.0	.34	[−.28, .75]		
**Peer-reviewed publication**					.30	.58
Yes	41	19.5	.25***	[.14, .36]		
No^c^	17	11.7	.30**	[.10, .48]		
Continuous moderators	*k*	Effect sizes	*B*	95% CI	*t-value*	*p*
Child age (months)	38	59	.04	[−.10, .17]	1.58	.30
Child sex (% boys)	37	57	−.03	[−.30, .24]	−.26	.81
Ethnic background (% white)	17	24	.14	[−.48, .76]	1.77	.40
Publication year	38	59	−.16	[−.25, −.06]	−3.56	.002
Quality assessment	38	59	−.03	[−.10, .04]	−.91	.38
Questionnaire reliability^d^	9	15	−.02	[−.27, .24]	−.16	.88

a Only categories with at least 4 df are included for the analysis.

b Only one category had df > 4 and the moderator analysis could not be reliably conducted.

c This category included book chapters and dissertations.

d Results are reported for the reliability of fathers’ questionnaire; results did not change when using the reliability of mothers’ questionnaires.

* *p* < .05,

** *p* < .01,

*** *p* < .001.

**TABLE 3:** Moderator analysis for correlation between parents.

Categorical moderators	*k*	*df*	*r*	95% CI	*Q_M_*	*p*
**Behavior type** ^a^					.96	.33
Global activation	2	1.0	.04*	[.01, .07]		
Challenging	18	8.1	.39**	[.21, .55]		
RTP	8	9.0	.34***	[.20, .47]		
**Behavior assessment method**					1.64	.20
Observation	17	8.1	.28**	[.08, .46]		
Questionnaire	16	4.4	.36***	[.22, .49]		
**Continent of data collection** ^b^					–	–
Asia	1	–	–	–		
Europe	13	3.0	.34*	[.01, .60]		
North America	9	6.8	.27**	[.04, .47]		
Oceania	5	1.0	.53	[−.99, .99]		
**Peer-reviewed publication** ^b^					–	–
Yes	25	10.3	.38***	[.02, .65]		
No^c^	8	2.8	.31*	[.17, .44]		
Continuous moderators	*k*	Effect sizes	*B*	95% CI	*t-value*	*p*
Child age (months)	14	28	.08	[−.16, .32]	3.17	.17
Child sex (% boys)	14	28	−.08	[−1.07, .91]	−.62	.63
Ethnic background (% white)	8	10	.13	[−.31, .57]	1.21	.35
Publication year	14	28	−.13	[−.23, −.01]	−3.10	.04
Quality assessment	14	28	.01	[−.04, .07]	1.95	.42
Questionnaire reliability^d^	7	16	.07	[−.12, .26]	2.87	.31

*Note*:

a Only categories with at least 4 df are included for the analysis.

b Only one category had df > 4 and the moderator analysis could not be reliably conducted.

c This category included book chapters and dissertations.

d Results are reported for the reliability of fathers’ questionnaire; results did not change when using the reliability of mothers’ questionnaires.

* *p* < .05,

** *p* < .01,

*** *p* < .001.

## Data Availability

Data and analysis code are available on OSF: https://osf.io/4tcgx/?view_only=ee01f3ff5b514ecf9b56523d189d8df7.

## Key Findings

- Activation parenting is known to benefit children by promoting healthy risk-taking and exploration of their environment, but has traditionally been conceptualized as a paternal practice.
- The first meta-analysis shows that, in partnered parents, fathers engage in slightly more activation parenting than mothers.
- The second meta-analysis shows that partnered mothers’ and fathers’ activation parenting behaviors are strongly correlated.

## Statement of Relevance

The present study examines the use of activation parenting by partnered mothers and fathers, a beneficial parental practice that aims to stimulate the child within reasonable boundaries. The meta-analytical results provide insight into gender differences and similarities in parental behaviors. Since both mothers and fathers engage in activation parenting, although fathers engage in more of it, intervention programs promoting this practice could be beneficial for families.

## References

[R1] Ainsworth MDS (1969). Ainsworth maternal scales. http://www.psychology.sunysb.edu/attachment/measures/content/ainsworth_scales.html

[R2] Aviv E, Waizman Y, Kim E, Liu J, Rodsky E, & Saxbe D (2025). Cognitive household labor: Gender disparities and consequences for maternal mental health and wellbeing. Archives of Women’s Mental Health, 28(1), 5–14. 10.1007/s00737-024-01490-w PMC11761833 38951218

[R3] Bakermans-Kranenburg MJ, Lotz A, Alyousefi-van Dijk K, & Van IJzendoorn M (2019). Birth of a father: Fathering in the first 1,000 days. Child Development Perspectives, 13(4), 247–253. 10.1111/cdep.1234 31894183 PMC6919930

[R4] Barth JM, & Parke RD (1993). Parent-child relationship influences on children’s transition to school. Merrill-Palmer Quarterly, 39(2), 173–195. https://www.jstor.org/stable/23090503

[R5] Bissonnette M (2019). Sur les traces du père sensible: Élaboration d’une grille d’observation de la sensibilité paternelle. [Unpublished doctoral dissertation]. Université de Sherbrooke, QC.

[R6] Bögels SM, & Perotti EC (2011). Does father know best? A formal model of the paternal influence on childhood social anxiety. Journal of Child and Family Studies, 20(2), 171–181. 10.1007/s10826-010-9441-0 21475711 PMC3048306

[R7] Borenstein M (2022). In a meta-analysis, the I-squared statistic does not tell us how much the effect size varies. Journal of Clinical Epidemiology, 152, 281–284. 10.1016/j.jclinepi.2022.02.013 36223816

[R8] Borenstein M, & Hedges LV (2019). Effect sizes for meta-analysis. In Cooper H, Hedges LV, & Valentine JC, (Eds.). The handbook of research synthesis and meta-analysis. (3rd ed., pp. 207–241). Russell Sage Foundation. 10.7758/9781610448864

[R9] Cabrera NJ, Fitzgerald HE, Bradley RH, & Roggman L (2014). The ecology of father-child relationships: An expanded model. Journal of Family Theory & Review, 6(4), 336–354. 10.1111/jftr.12054

[R10] Cabrera NJ, Hofferth SL, & Chae S (2011). Patterns and predictors of father–infant engagement across race/ethnic groups. Early Childhood Research Quarterly, 26(3), 365–375. 10.1016/j.ecresq.2011.01.001 22110258 PMC3220616

[R11] Cabrera N, Tamis-LeMonda CS, Bradley RH, Hofferth S, & Lamb ME (2000). Fatherhood in the twenty-first century. Child development, 71(1), 127–136. 10.1111/14678624.00126 10836566

[R12] Chen HH (2013). Couple relationship quality, coparenting, and fathering in Taiwan. Early Child Development and Care, 183(6), 827–842. 10.1080/03004430.2012.723443

[R13] Cooke JE, Deneault A-A, Devereaux C, Eirich R, Fearon RMP, & Madigan S (2022). Parental sensitivity and child behavioral problems: A meta-analytic review. Child Development, 93(5), 1231–1248. 10.1111/cdev.13764 35357693

[R14] Cox MJ, & Paley B (2003). Understanding families as systems. Current Directions in Psychological Science, 12, 193–196. 10.1111/1467-8721.01259

[R15] Crawley SB, & Sherrod KB (1984). Parent-infant play during the first year of life. Infant Behavior and Development, 7(1), 65–75. 10.1016/S0163-6383(84)80023-5

[R16] Deneault A-A, Cabrera NJ, & Bureau J-F (2022a). A metaanalysis on observed paternal and maternal sensitivity. Child Development, 93(6), 1631–1648. 10.1111/cdev.13832 35904112

[R17] Deneault A-A, Cabrera N, Ghosh R, Tölle AS, Seethaler J, Majdandžić M, & Reich SM (2022b). Challenging parenting behaviors in ethnically diverse two-parent families in the United States: Association with infants’ social competence and behavior problems. Early Childhood Research Quarterly, 58, 115–124. 10.1016/j.ecresq.2021.08.006 34658506 PMC8516126

[R18] Dickson KL (1994). The parent-infant communication system: Infant smiles in relation to play type and gaze direction. University of Utah.

[R19] Fabiano GA (2007). Father participation in behavioral parent training for ADHD: Review and recommendations for increasing inclusion and engagement. Journal of Family Psychology, 21(4), 683. 10.1037/0893-3200.21.4.683 18179340

[R20] Fagan J, Day R, Lamb ME, & Cabrera NJ (2014). Should researchers conceptualize differently the dimensions of parenting for fathers and mothers? Journal of Family Theory & Review, 6(4), 390–405. 10.1111/jftr.12044

[R21] Feinberg ME (2003). The internal structure and ecological context of coparenting: A framework for research and intervention. Parenting: Science and Practice, 3(2), 95–131. 10.1207/S15327922PAR0302_01 21980259 PMC3185375

[R22] Feldman JS, & Shaw DS (2021). The premise and promise of activation parenting for fathers: A review and integration of extant literature. Clinical Child and Family Psychology Review, 24(3), 414–449. 10.1007/s10567-021-00351-7 34059958

[R23] Feldman R (2023). Father contribution to human resilience. Development and Psychopathology, 35(5), 2402–2419. 10.1017/S0954579423000354 37039132

[R24] Fliek L, Daemen E, Roelofs J, & Muris P (2015). Rough-and-tumble play and other parental factors as correlates of anxiety symptoms in preschool children. Journal of Child and Family Studies, 24(9), 2795–2804. 10.1007/s10826-014-0083-5

[R25] Flanders JL (2008). Rough-and-tumble play and the development of externalizing behaviour. McGill University.

[R26] Forbes EE, Cohn JF, Allen NB, & Lewinsohn PM (2004). Infant affect during parent–infant interaction at 3 and 6 months: Differences between mothers and fathers and influence of parent history of depression. Infancy, 5(1), 61–84. 10.1207/s15327078in0501_3 16915346 PMC1550219

[R27] Funder DC, & Ozer DJ (2019). Evaluating effect size in psychological research: Sense and nonsense. Advances in Methods and Practices in Psychological Science, 2, 156–168. 10.1177/2515245919847202

[R28] Geiger B (1995). The new father: A study of attachment to primary caregiving fathers. State University of New York at Albany.

[R29] Gagnon Lafond K (2014). L’agencement des relations père-enfant et mère-enfant: liens avec le développement social d’enfants d’âge préscolaire de familles nucléaires. Université de Sherbrooke.

[R30] Ghosh RA (2024). Latino fathers’ motivations, parental play, parent and friend relationship support, and children’s socioe-motional development from early childhood to adolescence in racially-ethnically diverse families. University of Maryland.

[R31] Grossmann K, & Grossmann KE (2000). Parents and toddlers at play: Evidence for separate qualitative functioning of the play and the attachment system. The organization of attachment relationships: Maturation, culture, and context, (pp. 13–37).

[R32] Grossmann K, Grossmann KE, Fremmer-Bombik E, Kindler H, Scheuerer-Englisch H, & Zimmermann P (2002). The uniqueness of the child-father attachment relationship: Fathers’ sensitive and challenging play as a pivotal variable in a 16-year longitudinal study. Social Development, 11(3), 307–331. 10.1111/1467-9507.00202

[R33] Haas BE (1988). Parenting beliefs of mothers and fathers: An assessment of differences in families from two cultures. Michigan State University.

[R34] Hallgren KA (2012). Computing inter-rater reliability for observational data: An overview and tutorial. Tutorials in Quantitative Methods for Psychology, 8(1), 23–34. 10.20982/tqmp.08.1.p023 22833776 PMC3402032

[R35] Harrer M, Cuijpers P, Furukawa TA, & Ebert DD (2021). Doing meta-analysis with R: A hands-on guide. Chapmann & Hall/CRC Press. 10.1201/9781003107347

[R36] Harrer M, Cuijpers P, Furukawa T, & Ebert DD (2019). dmetar: Companion R package for the guide “Doing Meta-Analysis in R” (Version 0.0.9000) [R package]. Retrieved from http://dmetar.protectlab.org/

[R37] Harrington B (2022). The new dad: The career-caregiving conundrum. In Grau Grau M, las Heras Maestro M, & Riley Bowles H, (Eds.). Engaged fatherhood for men, families and gender equality. Contributions to management science. Springer. 10.1007/978-3-030-75645-1_11

[R38] Jacklin CN, DiPietro JA, & Maccoby EE (1984). Sex-typing behavior and sex-typing pressure in child/parent interaction. Archives of Sexual Behavior, 13(5), 413–425. 10.1007/BF01541427 6517683

[R39] Kerns KA, & Barth JM (1995). Attachment and play: Convergence across components of parent-child relationships and their relations to peer competence. Journal of Social and Personal Relationships, 12(2), 243–260. 10.1177/0265407595122006

[R40] Lamb ME (1977). Father-infant and mother-infant interaction in the first year of life. Child Development, 48(1), 167–181. 10.2307/1128896

[R41] Lazarus RS, Dodd HF, Majdandžić M, de Vente W, Morris T, Byrow Y, Bögels SM, & Hudson JL (2016). The relationship between challenging parenting behaviour and childhood anxiety disorders. Journal of Affective Disorders, 190, 784–791. 10.1016/j.jad.2015.11.032 26625090

[R42] Lazarus RS, McLellan LF, & Hudson JL (2018). Recalled challenging parenting behavior and anxiety in adulthood: An exploratory retrospective cohort study. Journal of Child and Family Studies, 27, 1216–1227. 10.1007/s10826-017-0919-x

[R43] Lee JY, Volling BL, & Lee SJ (2021). Testing the father–child activation relationship theory: A replication study with low-income unmarried parents. Psychology of Men & Masculinities, 22(3), 551. 10.1037/men0000301

[R44] Lin X, Xie S, & Li H (2019). Chinese mothers’ and fathers’ involvement in toddler play activity: Type variations and gender differences. Early Child Development and Care, 189(2), 179–190. 10.1080/03004430.2018.1542529

[R45] Lindsey EW, Mize J, & Pettit GS (1997). Differential play patterns of mothers and fathers of sons and daughters: Implications for children’s gender role development. Sex Roles, 37(9), 643–661. 10.1007/BF02936333

[R46] Lindsey EW, & Mize J (2001). Contextual differences in parent–child play: Implications for children’s gender role development. Sex Roles, 44(3), 155–176. 10.1023/A:1010950919451

[R47] Lüdecke D (2019). esc: Effect size computation for meta-analysis (Version 0.5.1) [R package]. Zenodo. https://zenodo.org/records/1249218

[R48] MacDonald K, & Parke RD (1984). Bridging the gap: Parent-child play interaction and peer interactive competence. Child Development, 55(4), 1265–1277. 10.2307/1129996 6488955

[R49] MacDonald K (1987). Parent-child physical play with rejected, neglected, and popular boys. Developmental Psychology, 23(5), 705. 10.1037/0012-1649.23.5.705

[R50] Madigan S, Deneault A-A, Duschinsky R, Bakermans-Kranenburg MJ, Schuengel C, Van IJzendoorn MH, Ly A, Fearon RMP, Eirich R, & Verhage ML (2024). Maternal and paternal sensitivity: Key determinants of child attachment security examined through meta-analysis. Psychological Bulletin, 150(7), 839–872. 10.1037/bul0000433 38709619

[R51] Majdandžić M, de Vente W, & Bögels SM (2016). Challenging parenting behavior from infancy to toddlerhood: Etiology, measurement, and differences between fathers and mothers. Infancy, 21(4), 423–452. 10.1111/infa.12125

[R52] Majdandžić M, de Vente W, Colonnesi C, & Bögels SM (2018). Fathers’ challenging parenting behavior predicts less subsequent anxiety symptoms in early childhood. Behaviour Research and Therapy, 109, 18–28. 10.1016/j.brat.2018.07.007 30077804

[R53] Majdandžić M, Möller EL, de Vente W, Bögels SM, & van den Boom DC (2014). Fathers’ challenging parenting behavior prevents social anxiety development in their 4-year-old children: A longitudinal observational study. Journal of Abnormal Child Psychology, 42, 301–310. 10.1007/s10802-013-9774-4 23812638

[R54] Martínez-Pastor J-I, Jurado-Guerrero T, Fernández-Lozano I, & Castellanos-Serrano C (2024). Caring fathers in Europe: Toward universal caregiver families? Gender, Work & Organization, 31(5), 1616–1638. 10.1111/gwao.12948

[R55] McGill BS (2014). Navigating new norms of involved fatherhood: Employment, fathering attitudes, and father involvement. Journal of Family Issues, 35(8), 1089–1106. 10.1177/0192513x14522247

[R56] Mellen HS (2002). Rough-and-tumble between parents and children and children’s social competence. Adelphi University.

[R57] Miller CJ (1987). Mother-infant and father-infant interaction behavior. American University.

[R58] Möller EL, Majdandžić M, & Bögels SM (2015). Parental anxiety, parenting behavior, and infant anxiety: Differential associations for fathers and mothers. Journal of Child and Family Studies, 24(9), 2626–2637. 10.1007/s10826-014-0065-7

[R59] Möller EL, Majdandžić M, de Vente W, & Bögels SM (2013). The evolutionary basis of sex differences in parenting and its relationship with child anxiety in Western societies. Journal of Experimental Psychopathology, 4(2), 88–117. 10.5127/jep.026912

[R60] Möller EL, Nikolić M, Majdandžić M, & Bögels SM (2016). Associations between maternal and paternal parenting behaviors, anxiety and its precursors in early childhood: A meta-analysis. Clinical Psychology Review, 45, 17–33. 10.1016/j.cpr.2016.03.002 26978324

[R61] National Heart, Lung, and Blood Institute. (n.d.). Quality assessment tool for observational cohort and cross-sectional studies. Accessed April 30, 2024. https://www.nhlbi.nih.gov/health-topics/study-quality-assessment-tools

[R62] Olofson EL, & Schoppe-Sullivan SJ (2022). Same behaviors, different outcomes: Mothers’ and fathers’ observed challenging behaviors measured using a new coding system relate differentially to children’s social-emotional development. Children, 9(5), 675. 10.3390/children9050675 35626852 PMC9139470

[R63] Page MJ, McKenzie JE, Bossuyt PM, Boutron I, Hoffmann TC, Mulrow CD, Shamseer L, Tetzlaff JM, Akl EA, Brennan SE, Chou R, Glanville J, Grimshaw JM, Hróbjartsson A, Lalu MM, Li T, Loder EW, Mayo-Wilson E, McDonald S, & Moher D (2021). The PRISMA, 2020 statement: An updated guideline for reporting systematic reviews. British Medical Journal, 372, n71. 10.1136/bmj.n71 33782057 PMC8005924

[R64] Pancsofar N, Vernon-Feagans L, & Family Life Project Investigators. (2010). Fathers’ early contributions to children’s language development in families from low-income rural communities. Early Childhood Research Quarterly, 25(4), 450–463. 10.1016/j.ecresq.2010.02.001 21057648 PMC2967789

[R65] Panter-Brick C, Burgess A, Eggerman M, McAllister F, Pruett K, & Leckman JF (2014). Practitioner review: Engaging fathers–recommendations for a game change in parenting interventions based on a systematic review of the global evidence. Journal of Child Psychology and Psychiatry, 55(11), 1187–1212. 10.1111/jcpp.12280 24980187 PMC4277854

[R66] Paquette D (2004a). Theorizing the father-child relationship: Mechanisms and developmental outcomes. Human Development, 47, 193–219. 10.1159/000078723

[R67] Paquette D (2004b). Dichotomizing paternal and maternal functions as a means to better understand their primary contributions. Human Development, 47(4), 237–238. 10.1159/000078726

[R68] Paquette D, & Bigras M (2010). The risky situation: A procedure for assessing the father-child activation relationship. Early Child Development and Care, 180(1–2), 33–50. 10.1080/03004430903414687

[R69] Paquette D, Carbonneau R, Dubeau D, Bigras M, & Tremblay RE (2003). Prevalence of father-child rough-and-tumble play and physical aggression in preschool children. European Journal of Psychology of Education, 18, 171–189. 10.1007/BF03173483

[R70] Paquette D, & Puentes-Neuman G (2024). Intervening with fathers and their children from the perspective of the activation relationship. In Osofsky JD, Fitzgerald HE, Keren M, & Puura K, (Eds.). WAIMH handbook of infant and early childhood mental health: Biopsychosocial factors. Vol. 1, Chapter 17 (pp. 275–291) Springer. 10.1007/978-3-031-48627-2

[R71] Pellegrini AD, & Smith PK (1998). Physical activity play: The nature and function of a neglected aspect of playing. Child Development, 69(3), 577–598. 10.1111/j.1467-8624.1998.tb06226.x 9680672

[R72] Pellegrini AD (2002). Rough-and-tumble play from childhood through adolescence: Development and possible functions. In Smith PK, & Hart CH, (Eds.). Blackwell handbook of childhood social development. (pp. 437–453). Blackwell Publishing.

[R73] Power TG, & Parke RD (1983). Patterns of mother and father play with their 8-month-old infant: A multiple analyses approach. Infant Behavior and Development, 6(4), 453–459. 10.1016/S0163-6383(83)90256-4

[R74] Pustejovsky JE, & Tipton E (2022). Meta-analysis with robust variance estimation: Expanding the range of working models. Prevention Science, 23(3), 425–438. 10.1007/s11121-021-01229-7 33961175

[R75] Pustejovsky J (2020). clubSandwich: Cluster-robust (sandwich) variance estimators with small-sample corrections (Version 0.5.8) [R package]. Retrieved from https://github.com/jepusto/clubSandwich

[R76] R Core Team. (2024). R: A language for statistical computing. R Foundation for Statistical Computing. Retrieved from https://www.R-project.org/

[R77] Revelle W (2026). psych (Version 2.3.3) [R package]. Retrieved from https://mirror.ibcp.fr/pub/CRAN/web/packages/psych/psych.pdf

[R78] Rodrigues M, Sokolovic N, Madigan S, Luo Y, Silva V, Misra S, & Jenkins J (2021). Paternal sensitivity and children’s cognitive and socioemotional outcomes: A meta-analytic review. Child Development, 92(2), 554–577. 10.1111/cdev.13545 33511634

[R79] Roopnarine JL, Talukder E, Jain D, Joshi P, & Srivastav P (1990). Characteristics of holding, patterns of play, and social behaviors between parents and infants in New Delhi, India. Developmental Psychology, 26(4), 667. 10.1037/0012-1649.26.4.667

[R80] Roopnarine JL, & Mounts NS (1985). Mother-child and father-child play. Early Child Development and Care, 20(2-3), 157–169. 10.1080/0300443850200205

[R81] Rosenthal R (1995). Writing meta-analytic reviews. Psychological Bulletin, 118, 183–192. 10.1037/0033-2909.118.2.183

[R82] Ross H, & Taylor H (1989). Do boys prefer daddy or his physical style of play?. Sex Roles, 20(1), 23–33. 10.1007/BF00288024

[R83] Schoppe-Sullivan SJ, & Fagan J (2020). The evolution of fathering research in the 21st century: Persistent challenges, new directions. Journal of Marriage and Family, 82(1), 175–197. 10.1111/jomf.12645

[R84] Schoppe-Sullivan SJ, Kotila LE, Jia R, Lang SN, & Bower DJ (2013). Comparisons of levels and predictors of mothers’ and fathers’ engagement with their preschool-aged children. Early Child Development and Care, 183(3–4), 498–514. 10.1080/03004430.2012.711596 23645966 PMC3640561

[R85] Schwartz CR (2013). Trends and variation in assortative mating: Causes and consequences. Annual Review of Sociology, 39, 451–470. 10.1146/annurev-sco-071312-145544

[R86] Schwarzer G, Carpenter JR, & Rücker G (2019). metasens: Advanced statistical methods to model and adjust for bias in meta-analysis (Version 0.4-0) [R package].

[R87] Sigle-Rushton W, Goisis A, & Keizer R (2013). Fathers and fatherhood in the European Union. In Cabrera NJ, & Tamis-LeMonda CS, (Eds.). Handbook of father involvement: Multidisciplinary perspectives. (2nd ed., pp. 81–96). Routledge.

[R88] StGeorge J, & Freeman E (2017). Measurement of father-child rough-and-tumble play and its relation to child behaviors. Infant Mental Health Journal, 38(6), 709–725. 10.1002/imhj.21676 29088498

[R89] Sullivan C (2003). The benefits of parent-child play for the social development of preschoolers with varying levels of anxiety problems. Concordia University.

[R90] Tanner-Smith EE, Tipton E, & Polanin JR (2016). Handling complex meta-analytic data structures using robust variance estimates: A tutorial in R. Journal of Developmental and Life-Course Criminology, 2(1), 85–112. 10.1007/s40865-016-0026-5

[R91] Tipton E, & Pustejovsky JE (2015). Small-sample adjustments for tests of moderators and model fit using robust variance estimation in meta-regression. Journal of Educational and Behavioral Statistics, 40(6), 604–634. 10.3102/1076998615606099

[R92] Van der Gaag N, Gupta T, Heilman B, Barker G, &Van den Berg W (2023). State of the world’s fathers: Centering care in a world in crisis. Equimundo.

[R93] Veritas Health Innovation. (2020). Covidence systematic review software. https://www.covidence.org

[R94] Viechtbauer W (2010). Conducting meta-analyses in R with the metafor package. Journal of Statistical Software, 36(3), 1–48. 10.18637/jss.v036.i03

[R95] Villafana BM (1997). Physical motor competence and the rough and tumble play of toddlers. University of Akron.

[R96] Volling BL, Stevenson MM, Safyer P, Gonzalez R, & Lee JY (2019). In search of the father infant activation relationship: A person-centered approach. In Volling BL, &Cabrera NJ, (Eds.). Monographs of the society of research in child development. (pp. 50–63). Wiley. PMC6625660 31303683

[R97] Walker HA (1995). Parent-infant dyads: Similarities and differences in patterns of interactions. University of Utah.

[R98] Weston MJ (1982). The effects of gender, sex-role type and temperament on the play behavior of parents and infants. New York University.

[R99] Wickham H (2016). ggplot2: Elegant graphics for data analysis. Springer.

[R100] Zaman W (2012). Parental styles of interaction during reminiscing and play: Relations to children’s attachment. Emory University.

