# Supplementary material for: Activation parenting in mothers and fathers: A systematic review and meta-analysis
Source: Infant Ment Health J. Author manuscript; Available in PMC 2026 Jul 1. (PMC13285669; doi:10.1002/imhj.70101)

**Supplemental Table 1.** Quality Assessment Items

| **Number** | **Item** |
| --- | --- |
| QA_1 | The goal of the study is clear. |
| QA_2a | The country where the recruitment was conducted is specified. |
| QA_2b | The year(s) when the study was conducted is specified. |
| QA_2c | The type of population studied is specified. |
| QA_3 | At least 50% of eligible or approached participants participated in the study. |
| QA_4a | Participants were recruited using the same recruitment strategy/setting. |
| QA_4b | Participants were recruited during the same time period. |
| QA_4c | If there were inclusion and exclusion criteria, they were specified prior to the participant selection. |
| QA_5a | Were parental behaviors measured at multiple time points? |
| QA_5b | If longitudinal, was the participant retention rate between time points at least 80%? |
| QA_6 | Were the parental behaviors measured on a continuous scale? |
| QA_7 | Were coders unaware about other study variables? |
| Overall | Global appraisal |

**Supplemental Table 2.** Overlapping Samples

| **Sample Name** | **Publications** | **Publication Selected for the Meta-Analysis** | **Reason for Selection** |
| --- | --- | --- | --- |
| Baby Books 2 | Deneault 2022  Ghosh 2025 | Deneault 2022  Ghosh 2025 | Different time points |
| Lazarus | Lazarus 2016  Majdandžić 2018 (Structure) | Lazarus 2016  Majdandžić 2018 (Structure) | Lazarus has larger N, Majdandžić reports rough-and-tumble play |
| Lindsey | Lindsey 2000  Lindsey 2001 | Lindsey 2001 | Reports more effect sizes |
| Majdandžić | Majdandžić 2016  Majdandžić 2018 (Fathers’)  Majdandžić 2018 (Structure) | Majdandžić 2016  Majdandžić 2018 (Structure) | Different age groups |
| Roopnarine | Roopnarine 1990  Roopnarine 1993 | Roopnarine 1990 | Larger N and more readily extractable information |

**Supplemental Table 3.** Quality Assessment for Included Studies

| **Study ID** | **QA_1** | **QA_2a** | **QA_2b** | **QA_2c** | **QA_3** | **QA_4a** | **QA_4b** | **QA_4c** | **QA_5a** | **QA_5b** | **QA_6** | **QA_7** | **Overall** | **Score** |
| --- | --- | --- | --- | --- | --- | --- | --- | --- | --- | --- | --- | --- | --- | --- |
| **Barth 1993** | High | High | Low | High | Low | High | High | Low | Low | N/A | Low | Low | Low | 0.73 |
| **Chen 2013** | High | High | Low | High | Low | Low | High | Low | Low | N/A | High | N/A | Low | 0.42 |
| **Deneault 2022** | High | High | High | High | Low | High | High | High | Low | N/A | High | High | High | 0.55 |
| **Dickson 1995** | High | High | Low | Low | Low | High | High | High | Low | N/A | Low | High | Low | 0.64 |
| **Flanders 2011** | High | High | High | Low | Low | High | High | High | Low | N/A | High | N/A | Low | 0.64 |
| **Fliek 2015** | High | High | Low | High | Low | High | High | Low | Low | N/A | High | N/A | High | 0.85 |
| **Forbes 2004** | High | High | Low | High | High | High | High | High | High | High | Low | High | High | 0.85 |
| **Gagnon Lafond 2014** | High | High | Low | Low | Low | High | High | Low | Low | N/A | High | Low | Low | 0.5 |
| **Geiger 1995** | High | High | Low | High | Low | Low | High | High | Low | N/A | High | Low | Low | 0.55 |
| **Ghosh 2025** | High | High | High | High | Low | High | High | High | Low | N/A | High | High | High | 0.85 |
| **Haas 1988** | High | High | Low | High | Low | Low | High | Low | Low | N/A | High | N/A | Low | 0.82 |
| **Jacklin 1984** | High | Low | Low | Low | Low | High | High | High | Low | N/A | High | Low | Low | 0.58 |
| **Kerns 1995** | High | Low | Low | High | Low | High | High | Low | Low | N/A | Low | High | Low | 0.45 |
| **Lamb 1977** | High | High | Low | Low | Low | High | High | High | High | High | Low | Low | Low | 0.54 |
| **Lazarus 2016** | High | Low | Low | High | Low | Low | High | Low | Low | N/A | High | N/A | High | 0.64 |
| **Lazarus 2018** | High | High | Low | High | Low | High | High | Low | Low | N/A | High | N/A | High | 0.64 |
| **Lee 2021** | High | High | High | High | Low | High | High | Low | Low | N/A | High | Low | High | 0.69 |
| **Lin 2019** | High | High | Low | Low | High | High | High | Low | Low | N/A | High | N/A | High | 0.5 |
| **Lindsay 1997** | High | Low | Low | High | High | High | High | Low | Low | N/A | Low | Low | High | 0.58 |
| **Lindsey 2001** | High | Low | Low | High | High | High | High | Low | Low | N/A | Low | High | High | 0.58 |
| **MacDonald 1984** | High | High | Low | High | Low | High | High | High | Low | N/A | Low | Low | Low | 0.33 |
| **MacDonald 1987** | High | Low | Low | Low | Low | High | High | Low | Low | N/A | Low | High | Low | 0.75 |
| **Majdandžić 2014** | High | High | Low | High | Low | High | High | High | Low | N/A | High | High | High | 0.77 |
| **Majdandžić 2016** | High | High | Low | High | Low | Low | High | High | High | High | High | High | High | 0.45 |
| **Majdandžić 2018** | High | High | Low | High | Low | Low | Low | Low | Low | N/A | High | N/A | High | 0.77 |
| **Mellen 2002** | High | High | Low | High | Low | High | High | Low | Low | N/A | Low | N/A | Low | 0.58 |
| **Miller 1987** | High | High | Low | High | Low | High | High | Low | Low | N/A | Low | High | High | 0.64 |
| **Moller 2015** | High | High | Low | High | Low | High | High | Low | Low | N/A | High | N/A | High | 0.67 |
| **Olofson 2022** | High | High | Low | High | Low | Low | High | High | Low | N/A | High | High | High | 0.42 |
| **Paquette 2003** | High | High | Low | High | Low | Low | High | High | Low | N/A | Low | Low | Low | 0.42 |
| **Power 1983** | High | Low | Low | High | High | High | High | Low | Low | N/A | Low | Low | Low | 0.75 |
| **Roopnarine 1985** | High | Low | Low | Low | High | High | High | Low | Low | N/A | Low | Low | Low | 0.25 |
| **Roopnarine 1990** | High | High | High | High | High | Low | High | High | Low | N/A | High | Low | High | 0.33 |
| **Ross 1989** | High | Low | Low | Low | Low | High | High | High | Low | N/A | Low | Low | Low | 0.33 |
| **Schoppe-Sullivan 2013** | High | High | Low | High | Low | Low | High | Low | Low | N/A | High | N/A | High | 0.42 |
| **Sullivan 2003** | High | High | High | High | Low | Low | Low | High | Low | N/A | High | Low | High | 0.58 |
| **Villafana 1997** | High | High | Low | High | Low | High | High | Low | Low | N/A | High | Low | High | 0.58 |
| **Volling 2019** | High | Low | Low | High | Low | Low | High | Low | Low | N/A | High | Low | High | 0.42 |
| **Walker 1995** | High | High | Low | High | Low | High | High | High | Low | N/A | High | High | Low | 0.67 |
| **Weston 1982** | High | High | Low | Low | Low | Low | High | Low | Low | N/A | Low | Low | Low | 0.25 |
| **Zaman 2012** | High | High | Low | High | Low | High | High | Low | Low | N/A | High | High | High | 0.67 |

**Supplemental Figure 1.** Funnel Plot of the Difference Between Fathers’ and Mothers’ Activation Parenting Levels


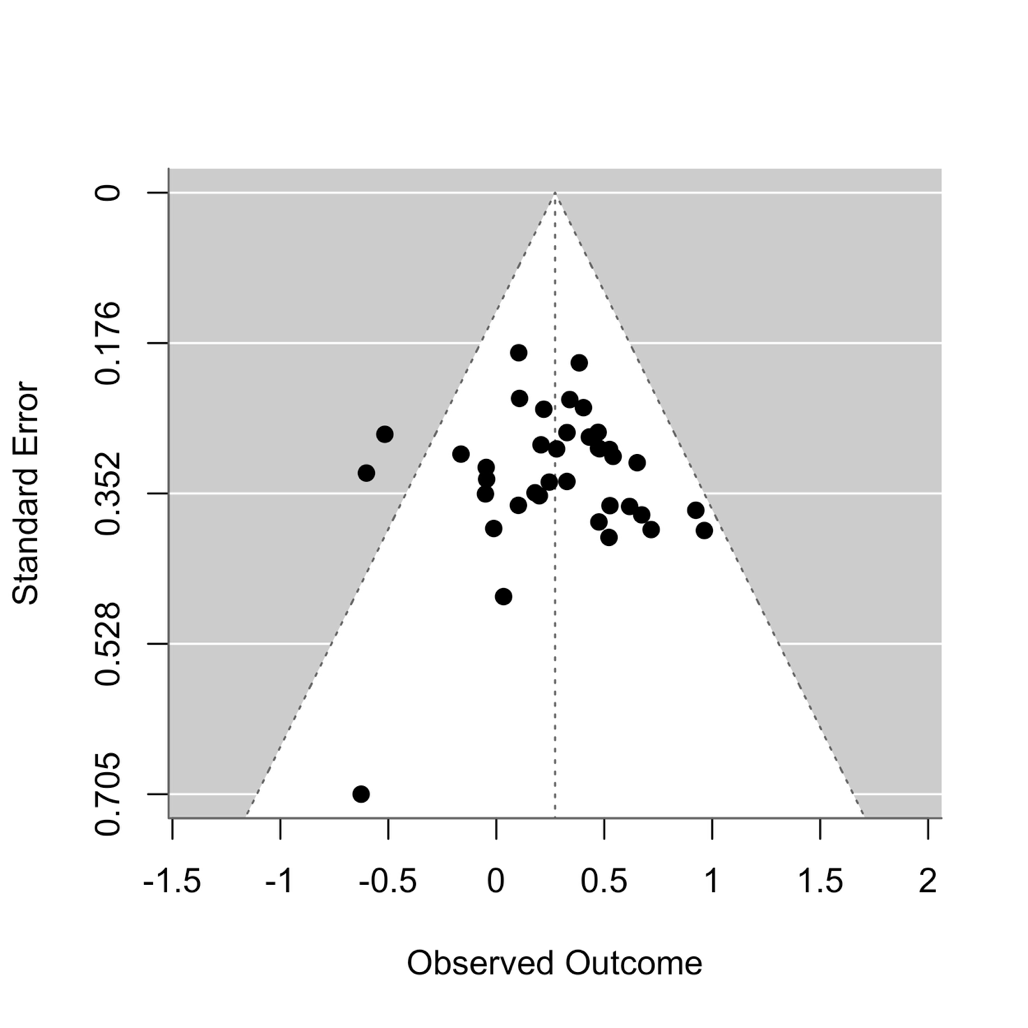


**Supplemental Figure 2.** Moderating Effect of Publication Year on the Difference Between Fathers’ and Mothers’ Activation Parenting Levels


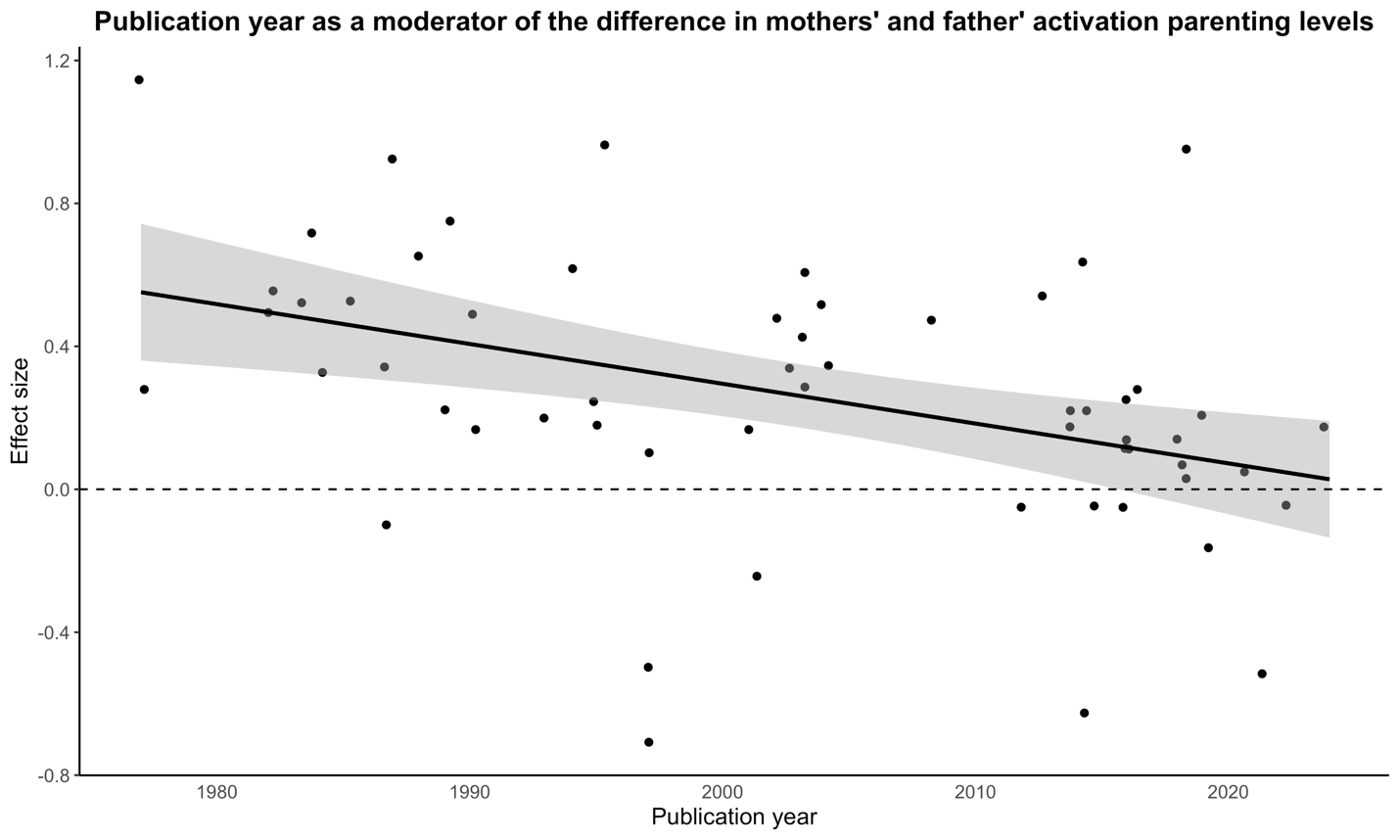


**Supplemental Figure 3.** Moderating Effect of Publication Year on the Difference Between Fathers’ and Mothers’ Activation Parenting Levels Across Behavior Types


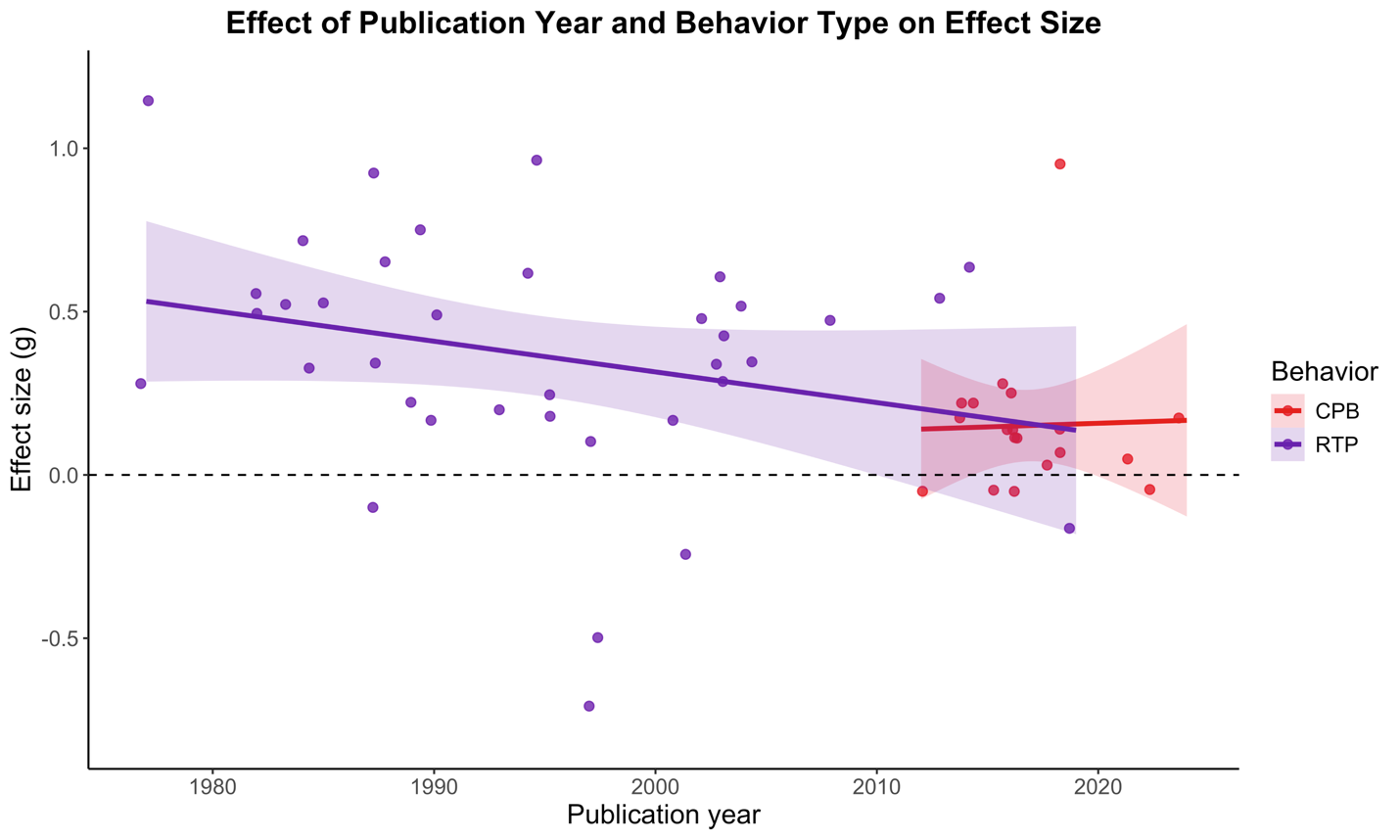


**Supplemental Figure 4.** Funnel Plot of the Correlation Between Fathers’ and Mothers’ Activation Parenting Levels


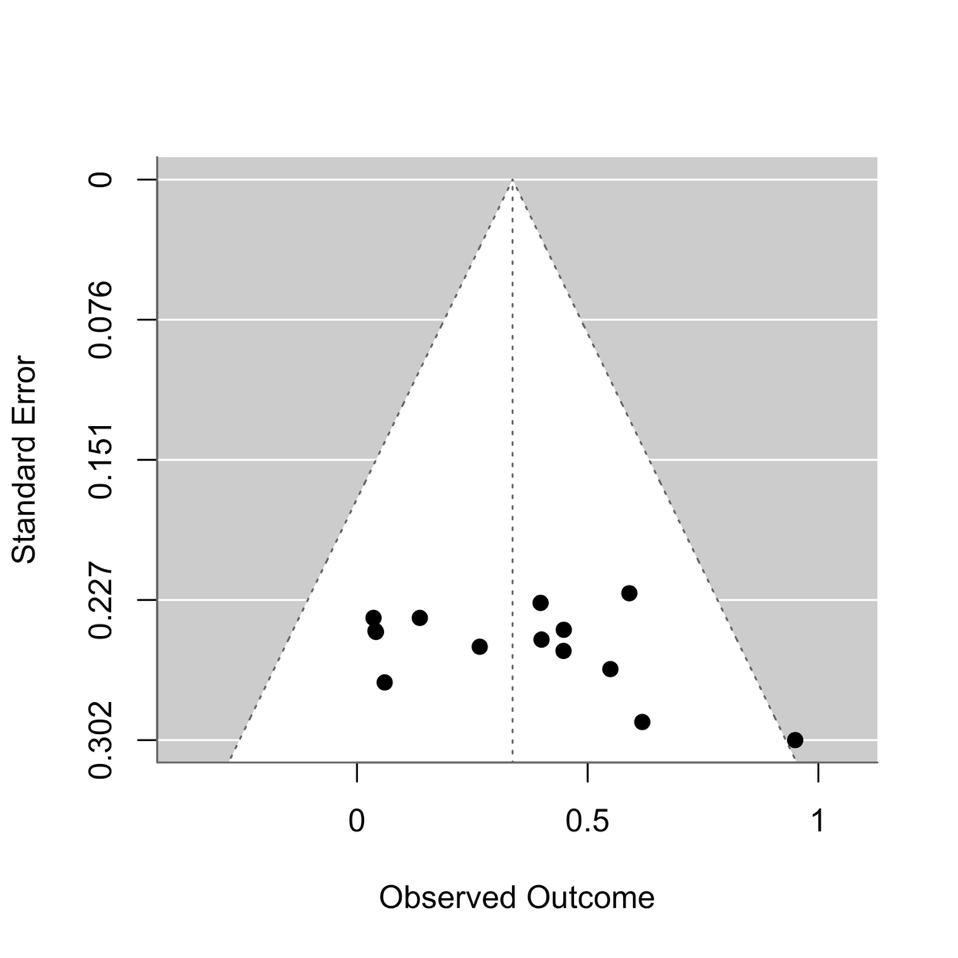


**Supplemental Figure 5.** Moderating Effect of Publication Year on the Correlation Between Fathers’ and Mothers’ Activation Parenting Levels


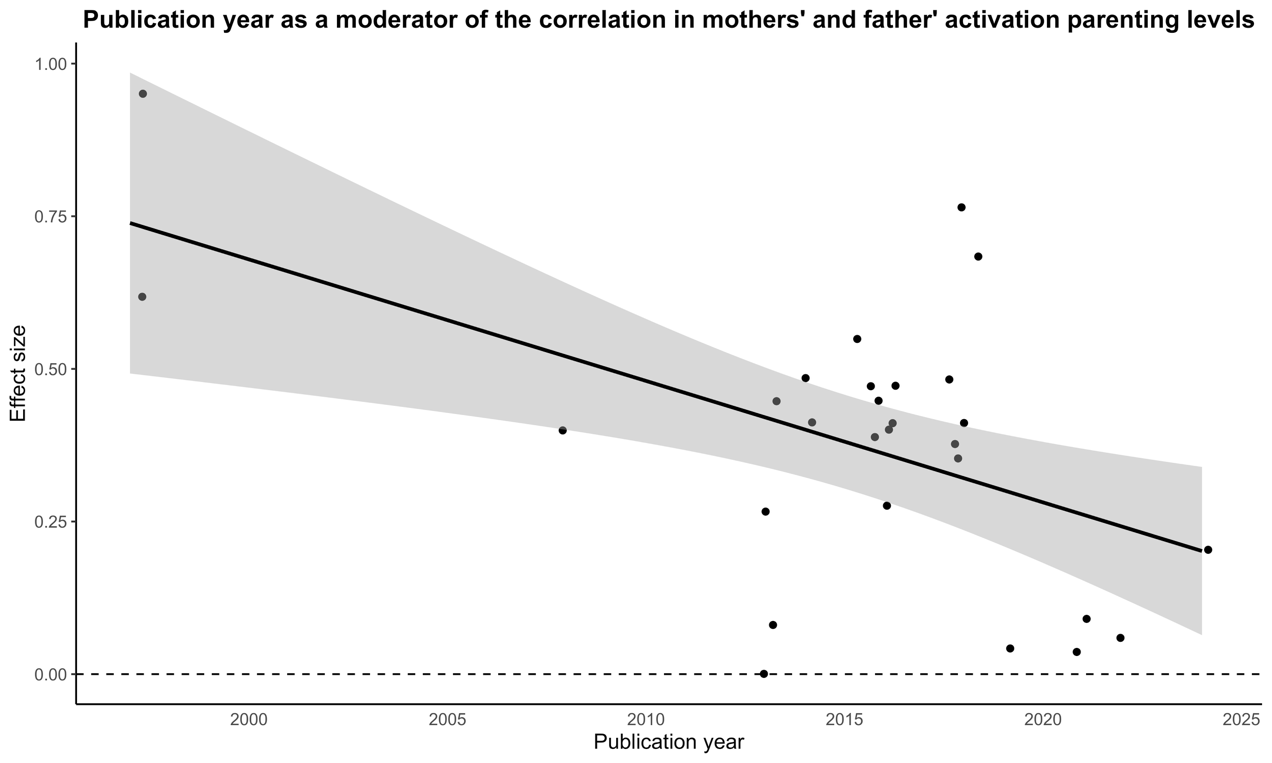

Supplement: Supplemental Material [file NIHMS2182192-supplement-Supplemental_Material.docx]
